# Elevated Nuclear PHGDH Synergistically Functions with cMyc to Reshape the Immune Microenvironment of Liver Cancer

**DOI:** 10.1002/advs.202205818

**Published:** 2023-04-20

**Authors:** Hongwen Zhu, Hua Yu, Hu Zhou, Wencheng Zhu, Xiongjun Wang

**Affiliations:** ^1^ CAS Key Laboratory of Receptor Research State Key Laboratory of Drug Research Shanghai Institute of Materia Medica Chinese Academy of Sciences Shanghai 201203 China; ^2^ Precise Genome Engineering Center School of Life Sciences Guangzhou University Guangzhou 510006 China; ^3^ Institute of Neuroscience State Key Laboratory of Neuroscience CAS Center for Excellence in Brain Science and Intelligence Technology Shanghai Institutes for Biological Sciences Chinese Academy of Sciences Shanghai 200031 China

**Keywords:** cMyc, CXCL1/IL8, liver cancer, neutrophil, nuclear PHGDH

## Abstract

Herein, we observed that nuclear localization of phosphoglycerate dehydrogenase (PHGDH) is associated with poor prognosis in liver cancer, and Phgdh is required for liver cancer progression in a mouse model. Unexpectedly, impairment of Phgdh enzyme activity exerts a slight effect in a liver cancer model. In liver cancer cells, the aspartate kinase‐chorismate mutase‐tyrA prephenate dehydrogenase (ACT) domain of PHGDH binds nuclear cMyc to form a transactivation axis, PHGDH/p300/cMyc/AF9, which drives chemokine *CXCL1* and *IL8* gene expression. Then, CXCL1 and IL8 promote neutrophil recruitment and enhance tumor‐associated macrophage (TAM) filtration in the liver, thereby advancing liver cancer. Forced cytosolic localization of PHGDH or destruction of the PHGDH/cMyc interaction abolishes the oncogenic function of nuclear PHGDH. Depletion of neutrophils by neutralizing antibodies greatly hampers TAM filtration. These findings reveal a nonmetabolic role of PHGDH with altered cellular localization and suggest a promising drug target for liver cancer therapy by targeting the nonmetabolic region of PHGDH.

## Graphical abstract

In this study, it is revealed that nuclear PHGDH cooperated with the powerful transcriptional factor cMyc, the acetyltransferase p300, and the super elongation factor AF9 to form a transactivation axis that drives chemokine *CXCL1* and *IL8* gene expression. The indicated chemokines urge the recruitment of tumor‐associated neutrophils and enhance tumor‐associated macrophage filtration in the liver, thereby advancing liver cancer.

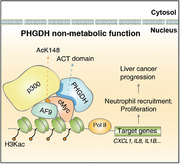

## 1 Introduction

Primary liver cancer, including hepatocellular carcinoma (HCC) and intrahepatic cholangiocarcinoma (ICC), is one of the major causes of cancer‐related death worldwide.^[^ ^1^ ^]^ Chemotherapy drugs are limited in availability and are usually less effective than surgery or transplantation.^[^ ^2^ ^]^ Genetic studies have uncovered several signaling pathways involved in hepatocarcinogenesis such as the Wnt/*β*‐catenin, MET, and YAP/Hippo pathways.^[^ ^3^, ^4^, ^5^ ^]^ A method combining hydrodynamic gene delivery and Sleeping Beauty (SB)‐mediated somatic integration has been widely used for long‐term gene expression in mouse hepatocytes to develop murine models for liver cancer research.^[^ ^6^ ^]^

In recent years, the omics field has been driven largely by technological advances, including RNA‐seq for transcriptomic analysis and mass spectrometry (MS), for rapidly advancing proteomic applications.^[^ ^7^ ^]^ Through the combined analysis of proteomics and RNA profiling, we found that two enzymes of the serine synthesis pathway (SSP), phosphoglycerate dehydrogenase (Phgdh) and phosphoserine aminotransferase 1 (Psat1), are upregulated in a mouse model of advanced‐stage liver cancer. SSP is a metabolic vulnerability in EGFR‐mutated cancer.^[^ ^8^ ^]^ PHGDH is the first rate‐limiting enzyme of the SSP and plays an important role in tumor resistance to serine starvation,^[^ ^9^ ^]^ reactive oxygen species (ROS) imbalance,^[^ ^10^ ^]^ sorafenib resistance,^[^ ^11^ ^]^ and brain metastasis.^[^ ^12^ ^]^ However, whether and how Phgdh regulates the progression of liver cancer is elusive.

Metabolic dysregulation reshapes tumor cells to enhance their growth and viability,^[^ ^13^ ^]^ but whether metabolic enzymes contribute to tumorigenesis through nonmetabolic functions is still relatively unclear. Some metabolic enzymes act independently of their classic metabolic activity to regulate tumor growth, survival, and metastasis.^[^ ^14^, ^15^ ^]^

In addition to hepatocytes, there are non‐parenchymal cells in liver tissue including macrophages, monocytes, neutrophils, lipid storage cells, and sinusoidal endothelial cells. Normally, the stroma is involved in maintaining tissue homeostasis; however, when hepatocytes become cancerous, the surrounding stroma changes to support tumor development.^[^ ^16^, ^17^ ^]^ Cancer cells, immune cells, and stromal cells, as well as their secreted factors, form the tumor microenvironment (TME).^[^ ^18^ ^]^ The communication between tumor cells and the surrounding TME is based on complex systemic networks. In addition to direct cell‐to‐cell contact, extracellular communications through secreted cytokines/chemokines play a key role in reprogramming the TME.^[^ ^18^ ^]^ Tumor cells have been shown to acquire the ability to produce growth‐promoting chemokines and to express chemokine receptors. For example, melanoma cells express several chemokines, including CXCL1/2/3, IL8, and CCL2/5, which have been implicated in tumor growth and progression.^[^ ^19^ ^]^ Herein, liver cancer cells expressed and secreted CXCL1/2, IL8, IL1B, and CCL5, most of which match the chemokines expressed by melanoma cells. We further proved that CXCL1/IL8 directed the recruitment of neutrophils and likely supported tumor‐associated macrophage (TAM) filtration. We speculate that reprogramming the TME by liver cancer cells facilitates cancer progression into an advanced stage.

Unlike the role of PHGDH in the cytoplasm, nuclear PHGDH mainly forms a complex with cMyc and synergistically drives gene expression required to recruit neutrophils and TAMs. Therefore, the liver cancer cells with nuclear PHGDH demonstrate a new function for metabolic enzymes in regulating the reciprocal action of tumor cells and the microenvironment independent of metabolic enzyme activity.

## 2 Results

### 2.1 Dynamic Intermodulation of the SSP and Cyto C Family Correlates with Liver Cancer Progression

**Figure 1:**
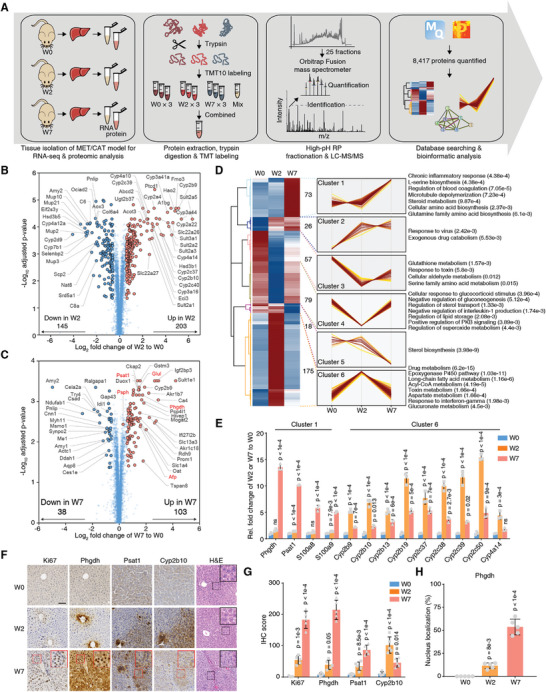
Dynamic intermodulation of the SSP and the Cyto C family correlates with liver cancer progression. A) Experimental workflow for quantitative proteomic analysis. B) The volcano plot presents the differentially regulated proteins in W2 versus W0. The *x*‐axis represents the log2‐transformed ratio in W2 relative to W0. The *y*‐axis represents the ‐log10‐transformed *p*‐value (adjusted by the Benjamini‐Hochberg method). C) The volcano plot presents the differentially regulated proteins in W7 versus W0. D) The differentially regulated proteins were separated into six clusters by HCA. Representative enriched biological processes for the proteins in each cluster are shown on the right. The *p*‐value in the bracket was calculated by Fisher's exact test. E) Validation of the indicated mRNA changes by qRT‐PCR in liver tissues of W0, W2, and W7 of the MET/CAT model. Rel., relative (mean ± SD, one‐way ANOVA followed by Dunnett's multiple comparisons test using W0 as the control, *n* = 3.) F) Immunohistochemistry (IHC) staining of Ki67, Phgdh, Psat1, and Cyp2b10, and H&E staining in mouse liver sections of W0, W2, and W7 of the MET/CAT model. Scale bar, 100 µm. G). The semi‐quantitative scoring (using a scale from 0 to 300) of indicated proteins was carried out (mean ± SD, one‐way ANOVA followed by Dunnett's multiple comparisons test using W0 as the control, *n* = 5). H) The semi‐quantitative scoring (using a scale from 0% to 100%) of nuclear‐localized Phgdh was carried out (mean ± SD, one‐way ANOVA followed by Dunnett's multiple comparisons test using W0 as the control, *n* = 5).

We performed integrative transcriptomic and proteomic analyses to explore the underlying mechanism of liver cancer progression. We systematically investigated the changes in C57BL/6 mouse liver tissues induced by human MET (hMET), a truncated *β*‐catenin mutant DN90‐*β*‐catenin (MET/CAT), and the SB transposase.^[^ ^20^ ^]^ Mice were sacrificed at weeks 0, 2, or 7 (W0, W2, or W7) after hydrodynamic injection, and the liver tissues were collected for RNA‐seq and tandem mass tag (TMT) 10‐plex‐based quantitative proteomic analysis (**Figure** **1**A). W2 was considered the initial stage and W7 was the final stage of malignant transformation in the liver. RNA‐seq identified 17 612 genes at all three‐time points, and 8417 proteins were quantified. Genes that overlapped between the transcriptomic and proteomic data (7751) were used for unbiased comparison (Figure S1A, Supporting Information). Protein reporter intensities in each TMT labeling channel were adjusted by median normalization to correct for differences in sample loading (Figure S1B, Supporting Information). The correlation matrix and principal component analysis suggested good reproducibility of sample quantification at the same time point and clear separation between time points (Figure S1C,D, Supporting Information). Correlation coefficients between the RNA and protein data were 0.63 in W2 and 0.39 in W7 (Figure S1E, Supporting Information).

Next, we analyzed the Kyoto Encyclopedia of Genes and Genomes (KEGG) pathways and Gene Ontology (GO) biological processes involved in the progression of MET/CAT‐driven hepatocarcinogenesis. Gene expression (RNA and protein) in W2 fluctuated more dramatically than in W7 (Figure S1F, Supporting Information). The most highly represented pathways and biological processes for the differentially expressed RNAs and proteins in W2 and W7 were liver detoxification‐related pathways or biological processes, including chemical carcinogenesis, oxidation‐reduction, and the epoxygenase P450 pathway, and metabolism‐related pathways such as steroid hormone biosynthesis and retinol metabolism (Figure S1G, Supporting Information). These results led us to focus on the dysregulation of detoxification‐related metabolism in liver cancer progression.

Proteins directly regulate biological behavior. Given the high degree of consistency between the RNA and protein data, we focused on the proteomic data to analyze the dynamic changes from W0 to W2 and then to W7 after SB induction. A total of 203 and 145 proteins were upregulated and downregulated, respectively, in W2 compared to W0. Members of the Cytochrome C family were considerably upregulated in W2 (Figure 1B). In W7, 103 and 38 proteins were up and downregulated, respectively. The clinically relevant liver cancer biomarkers Afp and Glul were upregulated in W7, suggesting the reliability of this liver cancer induction model. Notably, two key enzymes in l‐serine (Ser) biosynthesis, Phgdh and Psat1, considerably increased in W7. Another enzyme, Psph, also showed slight but significant upregulation (≈1.22‐fold) (Figure 1C). The differential protein expression analysis revealed that W2 and W7 represent two distinct states in liver cancer progression.

The differentially expressed proteins were analyzed by hierarchical cluster analysis (Figure 1D). Cluster 1 proteins were markedly upregulated in W7 and enriched for biological processes such as chronic inflammatory response, serine biosynthesis, microtubule depolymerization, steroid metabolism, and glutamine family amino acid biosynthesis. Interestingly, the Cluster 6 proteins were upregulated in W2 but had returned to basal levels in W7 and were associated with drug metabolism, the epoxygenase P450 pathway, acyl‐CoA metabolism, aspartate metabolism, and the interferon‐gamma response. Protein–protein interaction network analysis highlighted amino acid biosynthesis‐related proteins, including Phgdh, Psat1, Oat, and Glul, and inflammation‐related proteins, such as S100a8 and S100a9 in Cluster 1 and members of the cytochrome C family in Cluster 6 (Figure S1H, Supporting Information). S100a8 and S100a9 play pro‐tumorigenic roles in carcinogen‐induced HCC.^[^ ^21^ ^]^ The expression changes in Phgdh, Psat1, S100a8, S100a9, and a few members of the cytochrome C family were validated by quantitative real‐time PCR (qRT‐PCR) (Figure 1E). Immunohistochemistry confirmed the altered expression of Phgdh, Psat1, and Cyp2b10 (Figure 1F,G). As indicated by Ki67 intensity and hematoxylin‐eosin staining (H&E), liver cancer progression was associated with increased protein intensity and nuclear localization of Phgdh (Figure 1H). The SSP pathway was activated as increased serum serine levels (Figure S1I, Supporting Information).

This combination of proteomics and RNA‐sequencing revealed Phgdh in the SSP as a potential key driver of advanced liver cancer.

### 2.2 Conditional Deletion of Phgdh in Hepatocytes Attenuates MET/CAT‐Driven Hepatocarcinogenesis, but Inhibiting the Enzyme Activity of Phgdh does not Efficiently Prevent Liver Cancer Progression

**Figure 2:**
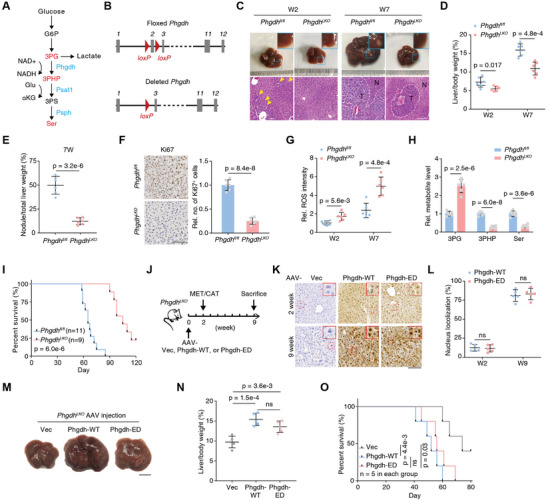
Loss of Phgdh activity does not efficiently hinder liver cancer progression induced by MET/CAT. A) Metabolic enzymes Phgdh, Psat1, and Psph and the related metabolites in the SSP. G6P, glucose‐6‐phosphate; 3PG, 3‐phosphoglycerate; 3PHP, 3‐phosphohydroxypyruvate; 3PS, 3‐phosphoserine; Ser, serine; nicotinamide adenine dinucleotide (oxidized form, NAD+; reduced form, NADH); Glu, glutamate; *α*KG, alpha‐ketoglutarate. B) Scheme of the *Phgdh* gene locus and related alleles. The *Phgdh* floxed alleles have two *loxP* sites (red triangles) flanking the second exon (gray boxes). Mice with *Phgdh* floxed alleles were crossed with a Cre line to generate the deleted *Phgdh* allele. C) Macroscopic images and H&E staining of the mouse (*Phgdh* ^*fl/fl*^ and *Phgdh^LKO^*) liver sections at W2 and W7 after hydrodynamic injection of MET/CAT constructs and the SB transposase. Scale bar, 100 µm. Yellow arrows indicate early neoplastic cells. The tumor region is outlined with a white dashed line. T denotes tumor, and N denotes non‐tumor. D,E) Liver/body weight ratios and tumor nodule/liver weight ratios of MET/CAT‐transfected mice (*Phgdh* ^*fl/fl*^ and *Phgdh^LKO^*) measured at W2 and W7 (mean ± SD, two‐tailed Student's *t*‐test, *n* = 6.). F) Ki67 staining of liver sections at week 7 after injection of MET/CAT (left panel). Relative numbers of Ki67‐positive cells in liver sections (right panel). Scale bars: 200 µm. Rel., relative; no., number (mean ± SD, two‐tailed Student's *t*‐test, *n* = 6). G) The relative ROS intensities were measured in liver samples of *Phgdh* ^fl/fl^ and *Phgdh^LKO^* mice at W2 and W7 after injection of MET/CAT. (Mean ± SD, two‐tailed Student's *t*‐test, *n* = 6.) H) The relative levels of three metabolites (3PG, 3PHP, and Ser) were measured in liver samples of *Phgdh* ^*fl/fl*^ and *Phgdh^LKO^* mice at W7 after injection of MET/CAT (mean ± SD, two‐tailed Student's *t*‐test, *n* = 6.) I) Kaplan–Meier plot showing the survival of *Phgdh* ^*fl/fl*^ and *Phgdh^LKO^* mice after injection of MET/CAT over 120 days (Log‐rank test). J) Schematic diagram of AAV treatment in MET/CAT‐induced liver tumor using *Phgdh^LKO^* mice. The AAV treatment (Vec, Phgdh‐WT, and Phgdh‐ED) was conducted in mice at W0. The mice were subjected to MET/CAT induction at W2 and sacrificed at W9. ED, enzyme‐dead. K) IHC staining of Phgdh in mouse liver sections at W2 and W9 after *Phgdh^LKO^* mice receiving AAV treatments (Vec, Phgdh‐WT, and Phgdh‐ED). Scale bar, 100 µm. L) The semi‐quantitative scoring (using a scale from 0% to 100%) of nuclear‐localized Phgdh was carried out (mean ± SD, two‐tailed Student's *t*‐test, *n* = 6). M) Macroscopic images of *Phgdh^LKO^* mouse livers under different AAV treatments (Vec, Phgdh‐WT, and Phgdh‐ED) at W9. Scale bar, 1 cm. N) Liver/body weight ratios of MET/CAT‐transfected mice under different AAV treatments (Vec, Phgdh‐WT, and Phgdh‐ED) at W9 were measured. (Mean ± SD, one‐way ANOVA followed by Tukey's multiple comparisons test, *n* = 5). O) Kaplan–Meier plot showing the survival of MET/CAT‐transfected mice under different AAV treatments (Vec, Phgdh‐WT, and Phgdh‐ED) (Log‐rank test, *n* = 5).

Phgdh and Psat1 are key enzymes that mediate the first two steps of serine (Ser) biosynthesis (**Figure** **2**A).^[^ ^22^ ^]^ To examine the functional roles of Phgdh in liver cancer, we induced liver tumorigenesis in *Phgdh* ^*fl/fl*^ (wild‐type, WT) and *Phgdh* ^*LKO*^ (liver‐specific knockout) mice by hydrodynamic injection. The *Phgdh* ^*LKO*^ mice were constructed by deleting the second exon of *Phgdh* (Figure 2B). PCR, western blotting, and IHC analyses of anti‐Phgdh and H&E staining confirmed the successful and specific knockout of *Phgdh* in mouse livers (Figure S2A–C, Supporting Information). At W2, the liver tissues of WT mice showed a slight increase in volume. H&E staining, outlined with a white line of dashes, showed scattered tumor cells in the liver of *Phgdh* ^*fl/fl*^ mice (Figure 2C, left). Compared with *Phgdh* ^*LKO*^ mice, we found a markedly increased liver volume and more tumor nodules in WT mice (*Phgdh* ^*fl/fl*^) at W7 after injection (Figure 2C, right). Loss of Phgdh in hepatocytes resulted in significant decreases in liver/body weight ratios and tumor nodules (Figure 2D,E), accompanied by decreased in vivo tumor cell proliferation (Figure 2F). In addition, Phgdh deletion considerably increased ROS levels in the liver (Figure 2G).^[^ ^10^ ^]^ To verify SSP dysfunction, we measured 3‐phosphoglycerate (3PG), 3‐phosphohydroxypyruvate (3PHP), and Ser by targeted liquid chromatography‐MS (LC‐MS) (Figure S2D, Supporting Information). Loss of Phgdh significantly blocked the SSP pathway, as evidenced by increased levels of 3PG and decreased levels of 3PHP and Ser (Figure 2H). As expected, the deletion of Phgdh in hepatocytes prolonged survival after MET/CAT‐driven hepatocarcinogenesis (Figure 2I). The tumors from *Phgdh* ^*LKO*^ mice showed less aggressiveness in tumor size, nodule number, Ki67‐positive cell number, and necrosis region (Figure S2E, Supporting Information). However, under normal living conditions, the liver/body weight ratios of 6‐week‐old *Phgdh* ^*LKO*^ and WT mice were comparable (Figure S2F–H, Supporting Information). Serum alanine aminotransferase (ALT) and aspartate aminotransferase (AST) levels did not differ in the livers of *Phgdh* ^*fl/fl*^ and *Phgdh* ^*LKO*^ mice (Figure S2I, Supporting Information), suggesting that liver function in the *Phgdh* ^*LKO*^ mice was normal.

To specifically characterize the enzymatic activity of Phgdh in MET/CAT‐induced tumorigenesis, adult *Phgdh^LKO^* mice were treated with adeno‐associated viruses (AAV) carrying the empty vector (Vec), wild‐type Phgdh (Phgdh‐WT), or enzyme‐dead Phgdh (Phgdh‐ED) under control of the thyroid hormone binding globulin promoter (Figure 2J). AAV treatment specifically and efficiently drove the expression of Phgdh‐WT or ‐ED in hepatocytes at week 2 (Figure 2K, upper), and the abolished PHGDH activity was confirmed (Figure S2J,K, Supporting Information). The mice were treated with MET/CAT to induce hepatocarcinogenesis with the accumulated nuclear PHGDH in tumor tissue (Figure 2K,L). Restoration of Phgdh‐WT in mouse liver promoted tumorigenesis, but the expression of Phgdh‐ED also facilitated this process to a similar extent (Figure 2M,N and Figure S2L, Supporting Information). Mice that received Phgdh‐ED exhibited a markedly reduced lifespan compared to the controls (Figure 2O). These results suggest Phgdh has another role in liver tumorigenesis independent of its enzymatic activity.

### 2.3 PHGDH Interacts with cMyc as a Coactivator Independent of its Enzyme Activity

**Figure 3:**
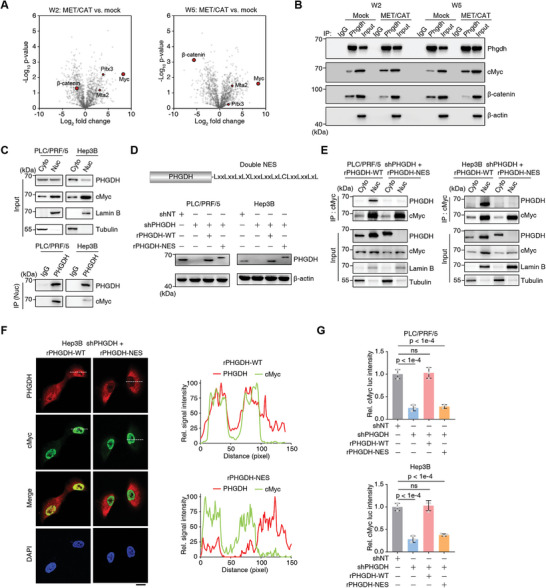
Phgdh interacts with cMyc independent of its enzyme activity. A) Volcano plot of the proteins differentially interacting with Phgdh as determined by the ratio between the MET/CAT‐induced liver cancer group and the mock group in W2 and W5. The mice in the mock group were injected with blank control plasmids. Transcriptional factors, including cMyc, were shown as brown red circles. B) Co‐immunoprecipitation (Co‐IP) assays were performed with an antibody against Phgdh followed by western blot analysis of Phgdh to detect the interaction between Phgdh and cMyc. Loading control, *β*‐actin. C) Separation of nuclei and cytosol was performed using PLC/PRF/5 and Hep3B cells. Co‐IP assay was conducted using nuclei to detect the interaction between Phgdh and cMyc. Cyto, cytosol; Nuc, nuclei. D) The construction of nuclear export sequence (NES) tagged PHGDH (PHGDH‐NES) in its C terminal was validated by immunoblotting in PLC/PRF/5 and Hep3B cells. shNT, a non‐targeting short hairpin RNA (shRNA); shPHGDH, shRNA against PHGDH; rPHGDH‐WT, shRNA‐resistant wild type PHGDH; rPHGDH‐NES, shRNA‐resistant PHGDH‐NES. Loading control, *β*–actin. E) Separation of nuclei and cytosol was performed using *PHGDH*‐depleted PLC/PRF/5 and Hep3B cells rescued with rPHGDH‐WT or rPHGDH‐NES. Co‐IP assay was conducted using nuclei to detect the interaction between Phgdh and cMyc. F) Immunofluorescence assay using *PHGDH*‐depleted Hep3B cells rescued with rPHGDH‐WT or rPHGDH‐NES. Cells were fixed with 80% methanol and stained with antibodies against cMyc or PHGDH. 4′, 6‐Diamidino‐2‐phenylindole (DAPI) was used as a nuclear localization marker (left panel). Scale bars: 20 µm. The fluorescence intensity profile of regions of interest (the dotted lines) was quantified to illustrate the colocalization of PHGDH and cMyc using ImageJ (right panel). G) cMyc transactivation was measured with a Dual‐Luciferase Reporter Assay System according to the manual using cells from (D) (mean ± SD, one‐way ANOVA followed by Dunnett's multiple comparisons test, *n* = 3).

To analyze the nonmetabolic effect of Phgdh, coimmunoprecipitation (Co‐IP) with LC‐tandem MS (MS/MS) was performed to identify proteins that interact with Phgdh in MET/CAT‐induced liver cancer. cMyc was one of the most enriched transcription factors driving HCC progression (**Figure** **3**A).^[^ ^23^, ^24^, ^25^ ^]^ Of note, the Biological General Repository for Interaction Datasets database also records cMyc as an interacting protein of Phgdh. Herein, cMyc interacted with Phgdh in the induced liver cancer group but not in the control group (Figure 3B).

Consistent with the results obtained in the induced mouse liver cancer model, PHGDH and cMyc interacted in human liver cancer cell lines independent of PHGDH enzyme activity (Figure S3A, Supporting Information). We stably knocked down *PHGDH* in these cell lines and observed the downregulation of the cMyc protein (Figure S3B, Supporting Information) and the specific interaction between PHGDH and cMyc (Figure S3C, Supporting Information). Luciferase assays showed that enzyme inactivation of PHGDH reduced the transactivation of cMyc by more than half (Figure S3D, Supporting Information).

We speculated that nuclear localization is a prerequisite for the interaction between PHGDH and cMyc. Extracted nuclei from two human liver cancer cell lines (PLC/PRF/5, Hep3B) showed partial localization of PHGDH (Figure 3C). Conversely, PSAT1, another SSP enzyme, showed no nuclear localization (Figure S3E, Supporting Information). To export PHGDH from nuclei to the cytosol; we artificially added a double nuclear export sequence (NES) to the PHGDH C‐terminus (Figure 3D). We found that PHGDH activity in PHGDH‐NES was similar to PHGDH‐WT (Figure S3F, Supporting Information). Co‐IP of separate cytosol and nuclear fractions showed that PHGDH‐NES successfully localized to the cytosol and did not interact with cMyc (Figure 3E). Immunofluorescence analysis showed that PHGDH and cMyc colocalized in the nucleus. Still, there was no colocalization with PHGDH‐NES (Figure 3F). Luciferase assay showed that PHGDH export from the nucleus decreased cMyc transactivation (Figure 3G). Two PHGDH NLS signal sequences were predicted, and we determined that NLS1 is more important for nuclear localization (Figure S3G,H, Supporting Information). In addition to the MET/CAT mouse model, we detected the expression and subcellular location of Phgdh in two other mouse models. MET/PIK3CA‐ and DEN‐induced hepatocarcinogenesis resulted in Phgdh upregulation and nuclear translocation (Figure S3I,J, Supporting Information). The evidence suggests that nucleus‐localized PHGDH synergistically functions with cMyc, independent of PHGDH activity.

### 2.4 The PHGDH‐ACT Domain is Required for the PHGDH/p300/cMyc/AF9 Axis and Regulates cMyc Transactivation

**Figure 4:**
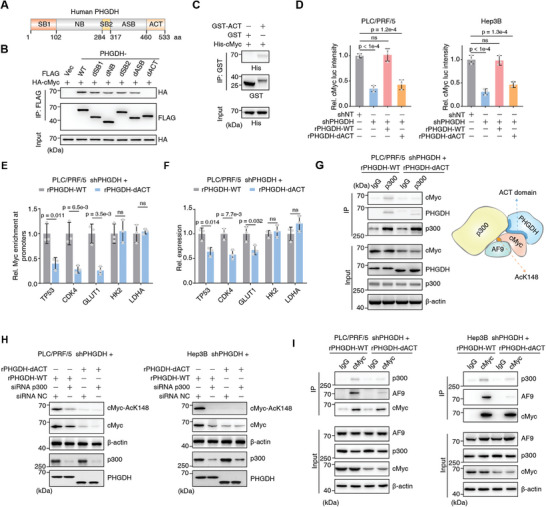
The PHGDH ACT domain is required for forming PHGDH/p300/cMyc/AF9 axis and regulates cMyc transactivation. A) Schematic diagram of human PHGDH protein and its five domains. aa, amino acid. B) HA‐tagged cMyc and FLAG‐tagged PHGDH (including WT and five domain truncates) were transiently transfected into HEK293T cells. Co‐IP was performed with an antibody against FLAG. Antibodies against HA and FLAG were used to detect the association between cMyc and PHGDH. HA was used as an input control. dSB1, SB1 domain depletion; dNB, NB domain depletion; dSB2, SB2 domain depletion; dASB, ASB domain depletion; dACT, ACT domain depletion. C) GST‐tagged ACT domain and His‐tagged cMyc protein were purified from *Escherichia* *coli*, and a GST pull‐down assay was performed by incubating both the recombinant proteins together. GST was used as a blank control. Antibodies against His and GST were used to detect the association between cMyc and the ACT domain. D) cMyc transcriptional activity was measured by the Dual‐Luciferase Reporter Assay System according to the manual using *PHGDH*‐depleted PLC/PRF/5 and Hep3B cells rescued with rPHGDH‐WT or rPHGDH‐dACT. Cells expressing shNT were used as control. rPHGDH‐dACT, shRNA‐resistant PHGDH‐dACT (mean ± SD, one‐way ANOVA followed by Dunnett's multiple comparisons test, *n* = 3). E) Chromatin immunoprecipitation (ChIP) analysis of cMyc enrichment on promoters of five canonical cMyc targets was performed using indicated cells. IgG was used as a blank control (mean ± SD, two‐tailed Student's *t*‐test, *n* = 3). F) qRT‐PCR assay of the five genes from (E) (mean ± SD, two‐tailed Student's *t*‐test, *n* = 3). G) Co‐IP analysis of p300, cMyc, and PHGDH was performed using *PHGDH*‐depleted PLC/PRF/5 cells rescued with rPHGDH‐WT or rPHGDH‐dACT. Antibody against p300 was used to enrich p300 associated complex. Immunoblotting analysis of PHGDH, cMyc, and p300 was performed using the indicated antibodies. H) p300 was transiently depleted by specific siRNA in *PHGDH*‐depleted PLC/PRF/5 cells rescued with rPHGDH‐WT or rPHGDH‐dACT. Immunoblotting analysis of cMyc‐AcK148, cMyc, p300, and PHGDH was performed using the indicated antibodies. NC, negative control. I) Co‐IP analysis of cMyc, p300, and PHGDH was performed using PHGDH‐depleted PLC/PRF/5 cells rescued with rPHGDH‐WT or rPHGDH‐dACT. Antibody against cMyc was used to enrich the cMyc‐associated complex. Immunoblotting analysis of PHGDH, p300, cMyc, and AF9 was performed using the indicated antibodies.

Human PHGDH contains five domains: substrate‐binding 1 (SB1), nucleotide‐binding, substrate‐binding 2 (SB2), allosteric substrate binding, and aspartate kinase‐chorismate mutase‐tyrA prephenate dehydrogenase (ACT) (**Figure** **4**A).^[^ ^26^ ^]^ To identify which region of PHGDH interacts with cMyc, we deleted each domain and performed Co‐IP assays. The ACT domain of PHGDH (aa 460–533) was required for its association with cMyc (Figure 4B).

The GST‐tagged ACT domain was purified from *Escherichia coli* while His‐tagged cMyc protein was expressed in *E. coli* as inclusion bodies, solubilized, refolded, and purified. GST pull‐down assays showed a significant interaction of cMyc with the PHGDH‐ACT domain (Figure 4C). The binding of the ACT domain to cMyc could be essential for PHGDH function in cMyc transactivation, as the capacity of PHGDH to transactivate cMyc was considerably diminished when we deleted the PHGDH‐ACT domain (Figure 4D). We selected five canonical downstream targets of c‐Myc and tested their expression in the absence of the PHGDH‐ACT domain. *TP53*, *CDK4*, and *GLUT1* were downregulated, while *HK2* and *LDHA* were unaffected suggesting PHGDH regulates a subset of c‐Myc targets (Figure 4E,F). Cells expressing the mutant PHGDH‐dACT also showed decreased cMyc protein expression (Figure S4, Supporting Information). Given that the ACT domain promotes cMyc transactivation and cMyc recruits p300 to activate gene expression, we speculated that the PHGDH‐ACT domain might associate with p300, but whether the ACT domain integrates p300 and cMyc together is still unclear. Co‐IP showed that in the absence of the ACT domain, p300 did not interact with cMyc and PHGDH (Figure 4G). As we observed a reduction of cMyc protein levels in cells expressing PHGDH‐dACT, we examined cMyc protein levels upon p300 depletion. We observed a concordant result, indicating that PHGDH and p300 affect cMyc in the same axis (Figure 4H). We hypothesized that the PHGDH‐ACT domain binds cMyc to form a signaling axis. We previously reported that AF9 recruits p300 to acetylate cMyc at K148^[^ ^27^ ^]^ and wondered whether the p300/cMyc/AF9 axis is associated with PHGDH. Compared with PLC/PRF/5 cells expressing WT PHGDH, cells expressing PHGDH‐dACT lost the p300/cMyc/AF9 axis, indicating that cMyc transactivation was impaired in cells lacking PHGDH‐ACT (Figure 4I). Combined with data from the interaction between the PHGDH‐ACT domain and cMyc, the transactivation of cMyc, and the formation of the p300/cMyc/AF9 axis via the PHGDH‐ACT domain, we conclude that PHGDH facilitates cMyc transactivation via its ACT domain to drive the formation of the p300/cMyc/AF9 axis.

### 2.5 The PHGDH/cMyc Axis Drives CXCL1/IL8 Expression

**Figure 5:**
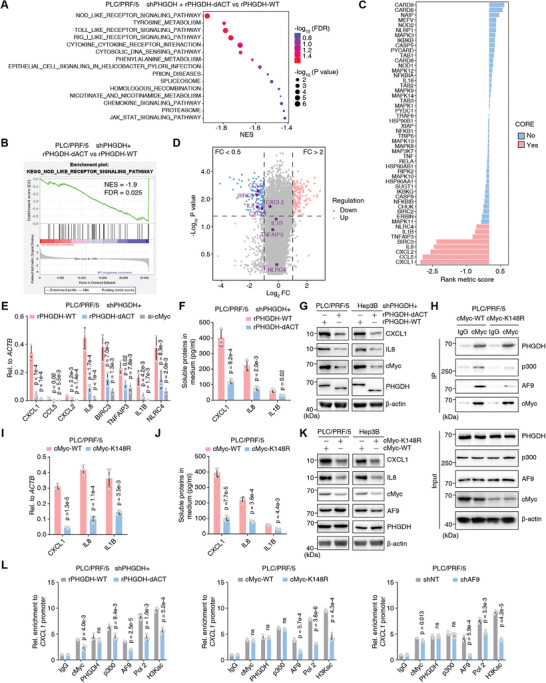
PHGDH/cMyc axis drives *CXCL1/IL8* expression. A–D) RNA‐sequencing analyses were performed using PHGDH‐depleted PLC/PRF/5 cells rescued with rPHGDH‐WT or rPHGDH‐dACT. Gene Ontology (GO) enrichment analyses of the differentially expressed genes were presented (A). GSEA enrichment plot of the KEGG pathway NOD‐like receptor‐related gene was shown in (B). The correlation of all NOD‐like receptor‐related gene expression with the phgdh expression status was displayed by the ranking metric score. A positive score indicates a correlation with the rPHGDH‐dACT and a negative score indicates a correlation with rPHGDH‐WT; The red indicates a gene that contributes most to the enrichment result and the blue indicates a gene that contributes less (C). The total differentially expressed genes (FC>2 or FC<0.5; *p* value <0.05) were displayed using a volcano plot. FC, fold change of rPHGDH‐dACT compared to rPHGDH‐WT (D). E) In *PHGDH*‐depleted PLC/PRF/5 cells with rPHGDH‐WT or rPHGDH‐dACT or further depletion of c‐Myc, qRT‐PCR validated the top up‐regulated genes from (C) (mean ± SD, one‐way ANOVA followed by Dunnett's multiple comparisons test, *n* = 3). F) ELISA examined the concentration of CXCL1/IL8 and IL1B in the medium culturing *PHGDH*‐depleted PLC/PRF/5 cells, which were rescued with rPHGDH‐WT or rPHGDH‐dACT (mean ± SD, two‐tailed Student's *t*‐test, *n* = 3). G) Immunoblotting analysis of CXCL1/IL8, PHGDH, and cMyc was performed using the indicated cells and antibodies. H) Co‐IP analysis of cMyc, p300, and PHGDH was performed using PLC/PRF/5 cells expressing WT or K148R mutant Myc. Antibody against cMyc was used to enrich the cMyc‐associated complex. Immunoblotting analysis of PHGDH, p300, cMyc, and AF9 was performed using the indicated antibodies. I) qRT‐PCR validated *CXCL1/IL8* and *IL1B* genes using PLC/PRF/5 cells expressing WT or K148R mutant Myc (mean ± SD, two‐tailed Student's t‐test, *n* = 3). J) ELISA examined the concentration of CXCL1/IL8 and IL1B in the medium culturing PLC/PRF/5 cells expressing WT or K148R mutant Myc (mean ± SD, two‐tailed Student's t‐test, *n* = 3). K) Immunoblotting analysis of CXCL1/IL8, PHGDH, and cMyc was performed using the indicated cells and antibodies. L) ChIP analysis of PHGDH, cMyc, p300, RNA Pol II (Pol 2), AF9, and H3Kac on *CXCL1* gene promoter was performed using indicated cells. IgG was used as a blank control (mean ± SD, two‐tailed Student's *t*‐test, *n* = 3).

To examine whether and how the oncogenic role of cMyc in the progression of liver cancer is regulated by PHGDH, we analyzed the RNA profiles of PLC/PRF/5 cells expressing WT or dACT PHGDH and GO enrichment analyses showed that the NOD‐like receptor‐related pathway ranked first among the differentially expressed genes (**Figure** **5**A). GSEA revealed that the KEGG pathway NOD‐like receptor‐related genes were downregulated in PLC/PRF/5 cells expressing PHGDH‐dACT versus WT (Figure 5B). The correlation of all NOD‐like receptor‐related gene expressions with PHGDH‐dACT was plotted by the ranking metric score. Red bars indicate genes that contribute most to the enrichment result, such as *CXCL1/2*, *CXCL8 (IL8)*, and *CCL5* (Figure 5C). All differentially expressed genes (FC > 2 or FC < 0.5; *p*‐value < 0.05) were displayed using a volcano (Figure 5D) and considerably downregulated genes, such as *CXCL1*, *IL8*, *CCL5*, and *BIRC3*, are marked. We tested the top eight genes contributing to GSEA enrichment on the NOD‐like receptor pathway by qRT‐PCR and observed a significant reduction in *CXCL1*, *IL8*, and *IL1B* expression after disruption of the PHGDH/cMyc interaction. Interestingly, cMyc knockdown (KD) further decreased the expression of these genes after PHGDH KD, suggesting PHGDH enhances the regulation of cMyc targets (Figure 5E and Figure S5A, Supporting Information). We then examined the soluble levels of CXCL1, IL8, and IL1B in the medium of cultured PLC/PRF/5 or Hep3B cells expressing WT or dACT and found that dACT reduced the concentration of secreted CXCL1, IL8, and IL1B in the medium, suggesting the decreased expression of CXCL1, IL8, and IL1B in liver cancer cells (Figure 5F and Figure S5B, Supporting Information). Immunoblotting verified the dramatic reduction of CXCL1 and IL8 after the loss of the PHGDH‐ACT domain (Figure 5G).

We previously reported that cMyc acetylation at K148 is required for the recruitment of AF9, which is a subunit of the super elongation complex and exacerbates proliferation in liver cancer cells.^[^ ^27^ ^]^ Herein, we examined the formation of the PHGDH/p300/cMyc/AF9 axis in the cells expressing WT or K148R cMyc. We verified that, compared to the interaction between PHGDH and WT cMyc, the reduced interaction between PHGDH and K148R cMyc is primarily related to the limited stability of the modified cMyc (Figure 5H). Accordingly, cells expressing K148R‐cMyc showed reduced expression of *CXCL1*, *IL8*, and *IL1B* (Figure 5I and Figure S5C, Supporting Information) and reduced secretion of CXCL1, IL8, and IL1B (Figure 5J and Figure S5D, Supporting Information). Immunoblotting analysis verified the dramatic reduction of CXCL1 and IL8 after the loss of cMyc protein stability (Figure 5K).

AF9 and PHGDH were involved in the p300/cMyc axis, so we tested whether AF9 depletion influenced *CXCL1* and *IL8* expression. Indeed, there was a reduction in secreted and total CXCL1 and IL8 protein, validating the PHGDH/p300/cMyc/AF9 axis, as dysregulation of each member individually caused the same effect on CXCL1 and IL8 expression (Figure S5E,F, Supporting Information).

Although subunits of the PHGDH/p300/cMyc/AF9 axis have the same effect on CXCL1 and IL8 expression, it remained unclear whether the axis directly targets the *CXCL1* and *IL8* gene promoter. A ChIP assay was performed using antibodies against PHGDH/p300/cMyc/AF9, Pol 2, and H3Kac to measure the direct regulation of *CXCL1* and *IL8* gene expression. In cells expressing PHGDH‐dACT, cMyc‐K148R, or AF9 depletion, the association of p300/cMyc/AF9, Pol II, and H3Kac with the *CXCL1* and *IL8* gene promoter was reduced to varying degrees, but the promoter of the PHGDH binding target gene was not affected (Figure 5L and Figure S5G, Supporting Information). *CSF1/3* are tumor immune‐remodeling genes that varied in the opposite direction, indicating a complicated regulatory circuit of inflammatory cytokines (Figure S5H, Supporting Information).

We conclude that the PHGDH/cMyc axis initiates *CXCL1* and *IL8* gene expression by recruiting p300 and AF9.

### 2.6 Destruction of PHGDH/cMyc Axis Hampered Tumor‐Associated Neutrophil/ Macrophage Recruitment by Liver Cancer Cells and Limited Liver Cancer Progression

**Figure 6:**
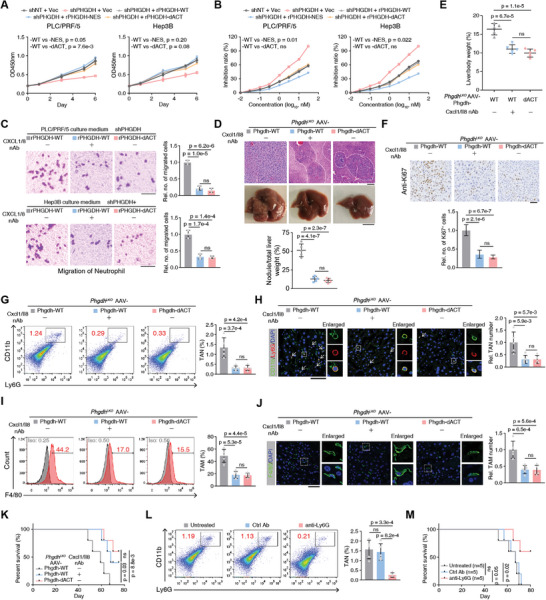
Destruction of the PHGDH/cMyc axis hampered neutrophil/macrophage recruitment by liver cancer cells and impaired liver cancer progression. A) Cell proliferation was evaluated by spectrofluorometer at wavelength OD450 in *PHGDH*‐depleted PLC/PRF/5 or Hep3B cells rescued with Vec, rPHGDH‐WT, rPHGDH‐NES, and rPHGDH‐dACT. Cells expressing shNT rescued with Vec were used as control (mean ± SD, two‐way ANOVA). B) Sorafenib inhibition was evaluated by trypan blue staining in *PHGDH*‐depleted PLC/PRF/5 or Hep3B cells rescued with Vec, rPHGDH‐WT, rPHGDH‐NES, and rPHGDH‐dACT. Cells expressing shNT rescued with Vec were used as control (mean ± SD, two‐way ANOVA). C) Neutrophil recruitment was evaluated by cell migration, which was performed by placing the medium from culturing *PHGDH*‐depleted PLC/PRF/5 or Hep3B cells in the lower well and neutrophil cells in the upper transwell chamber. Scale bars: 20 µm (mean ± SD, one‐way ANOVA followed by Tukey's multiple comparisons test, *n* = 3 per group). D) Macroscopic images and H&E staining of *Phgdh^LKO^* mouse livers under different AAV treatments (Phgdh‐WT with or without neutralized antibodies against Cxcl1/Il8 and Phgdh‐dACT) at W9 after hydrodynamic injection of MET/CAT constructs and the SB transposase. The tumor region is outlined with a white dashed line. Scale bars: 100 µm (upper); 1 cm (lower). The tumor nodule/liver weight ratios were measured (mean ± SD, one‐way ANOVA followed by Tukey's multiple comparisons test, *n* = 5 per group). E) Liver/body weight ratios of MET/CAT‐transfected mice under different AAV treatments (Phgdh‐WT with or without neutralizing antibodies (nAb) against Cxcl1/Il8 and Phgdh‐dACT) at W9 were measured (mean ± SD, one‐way ANOVA followed by Tukey's multiple comparisons test, *n* = 5 per group). F,H,J) IHC or IF staining of Ki67, Ly6G/CD11b, and F4/80 in mouse liver sections in (D). The relative number of Ki67^+^ cells (F), tumor‐associated neutrophil (TAN) (H), or tumor‐associated macrophage (TAM) (J) was pointed out by white arrows and calculated. Scale bars: 100 µm (F), 50 µm (H,J) (mean ± SD, one‐way ANOVA followed by Tukey's multiple comparisons test, *n* = 5 per group). G,I) Using fresh liver tumors in (D), representative flow cytometry data of neutrophils (CD11b^+^ Ly6G^+^) and TAMs (F4/80^+^) from indicated livers (mean ± SD, one‐way ANOVA followed by Tukey's multiple comparisons test, *n* = 5 per group). K) Kaplan–Meier plot showing the survival of MET/CAT‐transfected mice under different AAV treatments (Phgdh‐WT with or without neutralized antibodies against Cxcl1/Il8 and Phgdh‐dACT). (Log‐rank test, *n* = 5 per group). L) Using fresh liver tumors from the mice group Phgdh‐WT with or without neutralized antibodies against Ly6G, representative flow cytometry data of neutrophils (CD11b^+^ Ly6G^+^) from indicated livers (mean ± SD, one‐way ANOVA followed by Tukey's multiple comparisons test, *n* = 5 per group). M) Kaplan–Meier plot showing the survival of MET/CAT‐transfected mice under different treatments (Phgdh‐WT with or without neutralized antibodies against Ly6G). (Log‐rank test, *n* = 5 per group).

To test whether loss of nuclear PHGDH or ACT domain deletion affects liver cancer cells, we first validated cancer cell proliferation using PHGDH KD cells rescued with PHGDH‐NES or PHGDH‐dACT. Compared with the loss of full‐length PHGDH, both deletion of the ACT domain and restored expression of cytosolic PHGDH has no significant effect on liver cancer cell proliferation (**Figure** **6**A). Liver cancer cells expressing high levels of PHGDH are resistant to sorafenib.^[^ ^11^ ^]^ Herein, liver cancer cells expressing PHGDH‐dACT exhibited sorafenib sensitivity similar to the control cells. Still, cells with cytosolic PHGDH were more resistant, suggesting the PHGDH/cMyc axis does not affect sorafenib resistance (Figure 6B). Because nuclear PHGDH forms an axis with cMyc and AF9, we examined sorafenib resistance in cells expressing WT or K148R cMyc, or AF9 depletion. We observed no obvious difference consistent with the observations in the PHGDH mutants (Figure S6A,B, Supporting Information).

In addition to hepatocytes, there are non‐parenchymal cells in liver tissue, especially macrophages and neutrophils, which form an immune network to mediate immune cell infiltration in the tumor stroma and play a vital role in driving tumor progression.^[^ ^16^, ^17^ ^]^ CXCL1 and IL8, secreted by macrophages, monocytes, or other cells, direct neutrophil recruitment to the liver and promotes liver cancer progression.^[^ ^28^, ^29^, ^30^, ^31^, ^32^ ^]^ Compared to control liver cancer cells, antibody‐neutralized or genetically modified PHGDH‐dACT cells exhibited reduced neutrophil recruitment (Figure 6C). Similar results were obtained in cells expressing cMyc‐K148R or AF9 depletion (Figure S6C,D, Supporting Information). We restored WT and NLS1‐deleted (dNLS1) Phgdh under the thyroid hormone binding globulin promoter in *Phgdh^LKO^* mice and injected the mice with CAT/MET to initiate hepatocarcinogenesis. Loss of NLS1 inhibited nuclear localization of Phgdh in vivo without altering total protein levels (Figure S6E, Supporting Information). Consistent with previous results, Phgdh without NLS1 lost its pro‐tumor functions in both tumor volume and Cxcl1/Il8 expression (Figure S6F,G, Supporting Information).

To verify the role of PHGDH/cMyc axis‐regulated Cxcl1/Il8 expression in liver cancer progression, we delivered AAV carrying Phgdh‐WT or Phgdh‐dACT under the thyroid hormone binding globulin promoter into *Phgdh^LKO^* adult mice. Hepatocarcinogenesis was induced with MET/CAT, and neutralizing antibodies (nAbs) against Cxcl1/Il8 were then administered. As expected, the nAb‐treated Phgdh‐WT and Phgdh‐dACT groups showed fewer tumor nodules, which were ascertained by H&E staining and outlined with a white dashed line, than the untreated Phgdh‐WT group (Figure 6D). Mice from the nAb‐treated Phgdh‐WT and Phgdh‐dACT groups also showed lower liver/body ratios (Figure 6E). We restored Cxcl1 and Il8 to attempt to rescue the loss of Phgdh/cMyc axis‐regulated immune functions in liver cancer progression. We first validated Phgdh/cMyc axis‐regulated Cxcl1/Il8 expression in Hepa1‐6 cells (Figure S6H,I, Supporting Information). Phgdh‐WT or Phgdh‐dACT restored Phgdh KD Hepa1‐6 cells with or without Cxcl1 and Il8 overexpression (Figure S6J,K, Supporting Information). The cells were orthotopically transplanted into mouse livers, and examination showed that Cxcl1 and Il8 overexpression partially rescued the phenotype of a disrupted Phgdh/cMyc axis (Figure S6L,M, Supporting Information), verifying that the immune regulatory functions of Phgdh/cMyc axis are largely dependent on Cxcl1/Il8.

IHC staining of Ki67 suggested that loss of the Phgdh/cMyc axis or the neutralization of Cxcl1 and Il8 hampered cancer cell proliferation in the liver (Figure 6F). We also showed that neutralizing Cxcl1 and Il8 signals almost abolished neutrophil recruitment to the liver (Figure 6G,H). Thus, macrophage enrichment was reduced (Figure 6I,J), the proportion of filtrated T cells was slightly decreased (Figure S7A, Supporting Information), and survival was considerably prolonged (Figure 6K). We also directly depleted neutrophils by using a Ly6G depleting antibody (Figure 6L) and found that loss of neutrophils reduced not only the enrichment of TAMs in the liver but also prolonged survival (Figure 6M and Figure S7B, Supporting Information). These data demonstrate that the Phgdh/cMyc axis in liver cancer cells is required for recruiting neutrophils and TAM, which facilitates liver cancer progression.

### 2.7 Nuclear PHGDH Predicts Poor Prognosis and Synergistically Associates with cMyc in Clinical Patients

**Figure 7:**
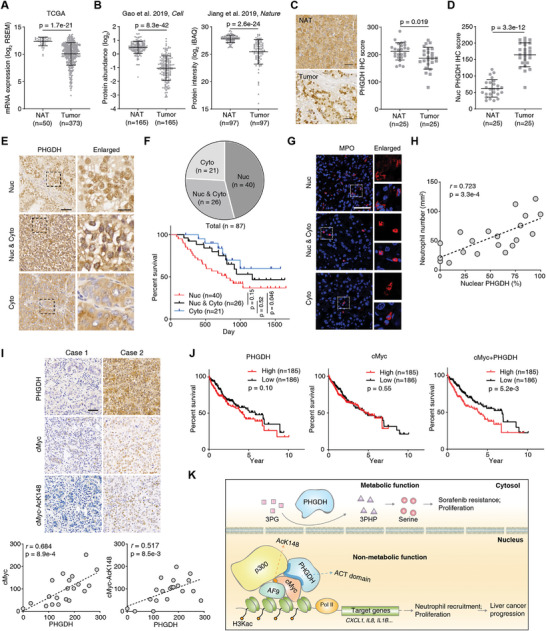
Nucleus‐localized PHGDH predicts poor prognosis and associates with cMyc in the clinic. A) Comparation of *PHGDH* expression levels in HCC tumors and normal adjacent tissues (NATs) using the TCGA dataset (Mann–Whitney test). B) Comparation of PHGDH protein levels in HCC tumors and paired NATs using the two HCC proteomic datasets (Gao *et al*. 2019, *Cell*; Jiang *et al*. 2019, *Nature*) (Wilcoxon matched‐pairs signed rank test). C,D) IHC staining of PHGDH in paired human liver tissues (NAT) and liver cancer tissues (*n* = 25, Wilcoxon matched‐pairs signed rank test). Representative images of PHGDH staining in paired samples were shown. Scale bars: 50 µm. E) IHC staining of PHGDH in human liver cancer tissues. Representative images of the nucleus (Nuc), cytoplasm (Cyto), and both nucleus and cytoplasm (Nuc & Cyto) localized PHGDH were shown. Scale bars: 50 µm (left); 10 µm (right). F) The patient counts of different subcellular localized PHGDH in human liver cancer tissues (upper). Kaplan–Meier plot showing the survival of patients with different subcellular signals of PHGDH. (Log‐rank test) (lower). G) IF analyses of infiltrated neutrophils in human liver cancer tissues. H) The semi‐quantitative scoring between nuclear PHGDH intensity (using a scale from 0% to 100%) and neutrophils (using a scale from 0 to 300) was carried out (Pearson product‐moment correlation test). I) The positive correlation of PHGDH and cMyc or PHGDH and cMyc‐AcK148 in clinical liver cancer patients were examined by IHC. The right panels show the semi‐quantitative scoring (using a scale from 0 to 300) between two staining signals carried out (Pearson product‐moment correlation test). Scale bar, 50 µm. J) Survival plot of PHGDH expression, cMyc expression, the combined expression of PHGDH, and cMyc in TCGA‐LIHC data with median cutoff. (Log‐rank test.) K) Work model of the nonmetabolic role of PHGDH.

To discover the clinical significance of PHGDH, we mined PHGDH expression in liver tumor tissue and normal adjacent tissues (NATs). The RNA and total protein levels of PHGDH were downregulated in tumor tissues (**Figure** **7**A,B). Generally, the downregulation of gene expression indicates that the gene would play a suppressive role in tumor development. However, this and a prior study^[^ ^11^ ^]^ confirmed that PHGDH contributes to liver cancer progression. We performed IHC using 25 paired liver tumor samples. We consistently found that total PHGDH was downregulated in tumor tissues. Still, its primary site of localization changes from the cytosol to the nuclear, with the intensity of nuclear PHGDH dramatically increased in tumor tissue (Figure 7C,D).

To validate the localization of PHGDH in human liver cancer tissues, we analyzed 87 tumor samples that showed positive signals. Based on the subcellular localization of the IHC signals of PHGDH, these tissues can be categorized into three types: nuclear (Nuc), both nucleus and cytoplasm (Nuc & Cyto), and cytoplasm localized (Cyto) (Figure 7E). Notably, about half of the samples showed strong nuclear localization of PHGDH (Figure 7F, upper panel). Patients with strong nuclear PHGDH had a poorer prognosis than patients with primarily cytoplasmic PHGDH (Figure 7F, lower panel). More importantly, in line with our findings in a mouse model and human cancer cell lines, neutrophil infiltration in human HCC samples positively correlates with the nuclear PHGDH signal (Figure 7G,H).

To verify whether PHGDH and cMyc have synergistic effects in the clinical setting, we performed IHC with clinical liver cancer samples using antibodies against PHGDH, cMyc, and cMyc‐AcK148. We observed a positive correlation between PHGDH and cMyc, and PHGDH and cMyc‐AcK148, suggesting PHGDH should positively regulate cMyc protein levels in clinical (Figure 7I). We also mined the TCGA data and evaluated the association of PHGDH, cMyc, and PHGDH/cMyc expression with patient survival and found that the combined expression of PHGDH and cMyc is a significant prognostic indicator (Figure 7J). To validate the PHGDH/cMyc/AF9/P300 axis in clinical samples, we performed endogenous Co‐IP using PHGDH antibody and found that cMyc, AF9, and p300 were associated with PHGDH in advanced HCC (Figure S8, Supporting Information). Thus, the translocation of PHGDH from the cytoplasm to the nucleus is an unfavorable biomarker in patients with liver cancer. These findings suggest that PHGDH and cMyc exert synergistic effects on liver cancer progression.

## 3 Discussion

Herein, we discovered a distinct function of PHGDH, which promotes cMyc transactivation via the ACT domain to drive promoter expression of genes required for neutrophil recruitment and TAM enrichment in liver cancer tissue, driving liver cancer advancement (Figure 7K).

Integrative transcriptomic and proteomic analyses are valuable tools for investigating biological issues.^[^ ^33^, ^34^ ^]^ We combined these two omics methods to examine the molecular events underlying MET/CAT‐induced liver carcinogenesis and identified the SSP through dynamic proteome profiling, supporting the validity of our study.

As summarized elsewhere,^[^ ^6^ ^]^ hydrodynamic transfection of MET/CAT with SB is a reliable and widely used method to generate liver cancer in mice. This method has been used in many studies to explore hepatocarcinogenesis.^[^ ^35^, ^36^, ^37^ ^]^ Here, we found that genes in the Afp, Glul, and cytochrome C families were gradually upregulated in a mouse model of liver cancer progression (Figure 1B,C). More importantly, two SSP enzymes, Phgdh and Psat1, showed robust upregulation. Given the importance of the SSP, in which Phgdh is the first rate‐limiting enzyme, we specifically deleted an exon of *Phgdh* in mouse livers. We performed tissue‐specific deletion because systemic knockout of *Phgdh* is lethal.^[^ ^9^ ^]^ To ensure the reliability of our MET/CAT‐induced liver cancer model, we tested the activity of AST and ALT in the livers of *Phgdh^LKO^* mice. We found no significant differences compared to control mice, indicating that loss of Phgdh does not cause abnormal liver function in the absence of MET/CAT.

We explored Phgdh interaction networks to characterize its nonmetabolic role and found that Phgdh associates with cMyc during different stages of MET/CAT‐driven liver carcinogenesis. Other reports have demonstrated that metabolic enzymes can regulate the transactivation of transcription factors. For example, FBP1 interacts with and inhibits HIF1*α*,^[^ ^15^ ^]^ while PKM2 interacts directly with the HIF1*α* and promotes transactivation of HIF1*α* target genes.^[^ ^38^ ^]^ Identifying cMyc as an interactor of PHGDH is a key step in this study. cMyc directly regulates more than one thousand target genes that affect many aspects of tumor behavior such as proliferation, growth, metastasis, metabolic abnormality, and drug resistance.^[^ ^39^ ^]^

The nuclear colocalization of PHGDH and cMyc is the basis for their interaction. We further detected the localization of PHGDH in clinical human liver cancer samples and found that 66 of 87 samples showed nuclear PHGDH signals. More considerably, nuclear localization of PHGDH correlated with a poorer prognosis, suggesting that PHGDH functionally contributes to tumor progression in a location‐dependent manner. Metabolic enzymes enter the nucleus to regulate gene expression or mRNA stability. Our previous study showed that UGDH localizes to the nucleus and binds HuR, eliminating UDP‐glucose‐mediated inhibition of HuR.^[^ ^40^ ^]^ Recently, a report demonstrated PHGDH enters the nucleus in pancreatic cancer under nutrient stress and exhibits an alternative activity.^[^ ^41^ ^]^ Thus, our study first uncovered a non‐metabolic role of nuclear PHGDH.

PHGDH facilitated the expansion of cMyc gene targeting such as CXCL1/2/5, IL8, IL1B, and CCL5. All of these chemokines can recruit neutrophils in the TME. Chemokines indirectly modulate tumor growth through their effects on tumor stromal cells and immune cells by inducing the release of growth and angiogenic factors from cells in the TME. Tumor cells and other cells in the TME acquire the ability to produce growth‐promoting chemokines, which form networks with chemokine receptors. Understanding the interplay between chemokine–chemokine receptor networks between tumor cells and their microenvironment is a novel approach to overcoming the problem of metastatic heterogeneity. Recent advances in understanding chemokine networks pave the way for developing a targeted therapeutic strategy to treat advanced cancer. Previous studies have identified that hepatic CXCL1/5 expression contributes to neutrophil infiltration in the liver, promoting steatosis‐to‐NASH progression in HFD‐fed mice^[^ ^42^ ^]^ or advanced stages of liver cancer.^[^ ^43^ ^]^ Blockade of these chemokine–chemokine receptor networks considerably improved outcomes in liver diseases.^[^ ^44^, ^45^ ^]^ Genetically engineered mouse models of non‐small‐cell lung cancer were used to demonstrate that neutrophil recruitment determines the nature of the tumor,^[^ ^46^ ^]^ suggesting that classification of liver cancer stages could also be achieved by examining neutrophil infiltration in the liver.

It is worth noting that some chemokines, such as IL8, have a dual role in promoting or suppressing cancer. Elevated tumor levels of IL8 have been detected in tumor progression, suggesting it may function as a mitogenic and angiogenic factor.^[^ ^47^, ^48^ ^]^ Dying cancer cells can be immunogenic and direct the antitumor immune response. IL8, for example, increases the immunogenicity of dying cancer cells by translocating calreticulin to the cell surface.^[^ ^49^ ^]^

Although cMyc controls many aspects of tumorigenesis, efforts to use cMyc as a therapeutic target has been frustrating, indicating some mysteries remain in our understanding of cMyc transactivation.^[^ ^50^, ^51^, ^52^ ^]^ Our study suggests the potential utility of cMyc transactivation by disrupting the PHGDH/cMyc axis in liver cancer cells. When we analyzed the expression of PHGDH and cMyc, we found their genes exhibit strong clinical synergy and can predict survival in patients with liver cancer.

Consistent with the evidence that PHGDH localizes to the nucleus at the cellular level, we also observed the nuclear localization of PHGDH advanced liver cancer in both human and mouse models. The nuclear intensity of PHGDH could mark the progression of liver cancer, and the PHGDH/cMyc axis represents a new layer of metabolic enzyme regulation of the reciprocal action of tumor cells and the microenvironment independent of PHGDH activity.

## 4 Experimental Section

### Antibodies and Reagents

Rabbit polyclonal or monoclonal antibodies against PHGDH (PA5‐82863, 1:50 for ChIP), PSAT1 (PA5‐22124, 1:200 for IHC), CD68 (14‐0688‐82, 1:500 for IHC), Ki67 (MA5‐14520, 1:500 for IHC), and Lamin B (33‐2000, 1:1000 for IB) and mouse monoclonal antibody against PHGDH (MA5‐31357, 1:200 for IHC, 1:1000 for IB, 1:100 for IP), human CXCL1 (MA5‐23811, 1:1000 for IB, 1:200 for ELISA), mouse Cxcl1 (MA5‐23745, 1:1000 for IB, 1:200 for ELISA), human IL8 (M801, 1:1000 for IB, 1:200 for ELISA), IL1B (BMS224‐2, 1:200 for ELISA), Ly6G (14‐5931‐82, 1:100 for IF), CD11b (53‐0112‐82, 1:100 for IF), and CD68 (MA5‐13324, 1:100 for IF) were purchased from Thermo Fisher. The antibody (ab197016) and ELISA kit (ab234567) against mouse Il8 were purchased from Abcam. The mouse monoclonal antibody against FLAG (F3165, 1:5000 for IB, 1:100 for IP) and the rabbit polyclonal antibody against AF9 (HPA001824, 1:1000 for IB, 1:100 for ChIP) were purchased from Sigma‐Aldrich. Antibody against HA (#3724, 1:2000 for IB) was purchased from Cell Signaling Technology (CST). Mouse monoclonal antibodies against *β*‐actin (60008‐1‐Ig, 1:5000 for IB), GST tag (66001‐2‐Ig, 1:5000 for IB, 1:100 for IP), and His tag (66005‐1, 1:3000 for IB) was purchased from Proteintech. Mouse monoclonal antibodies against cMyc (sc‐40, 1:2000 for IB, 1:200 for IHC, and 1:50 for ChIP), tubulin (sc‐5286, 1:2000 for IB), and CYP2B10 (sc‐73546, 1:50 for IHC) were purchased from Santa Cruz Biotech. A histone H3Kac antibody (Clone 2G1F9, 1:200 for ChIP, a POL2 antibody (Clone 4H8, 1:100 for ChIP), and a p300 antibody (Clone NM11, 1:200 for ChIP, 1:1000 for IB, 1:100 for IP) were purchased from Active Motif. The antibody against cMyc‐AcK148 was prepared in a previous study.^[^ ^27^ ^]^ siRNAs targeting human EP300 (106 443) were purchased from Thermofisher.

### Experimental Mice

The *Phgdh^LKO^* (*Phgdh^fl/fl^*:*Alb‐Cre*) mouse line was generated on a mixed FVB/N and C57BL/6 background by breeding *Phgdh^fl/fl^* mice with *Albumin‐Cre* transgenic mice. C57BL/6 mice were used for MET/CAT model construction in the proteomic study. All animal studies were conducted on male mice aged 6–24 weeks. The mice were group‐housed (4–5 mice per cage) except for fewer than 5% of the mice, which were single‐housed later because of the death of cage mates. All mice were maintained under a 12‐h light/dark cycle with free access to water and standard mouse chow. All mice received humane care, and all experimental procedures were approved by the Institutional Animal Care and Use Committee (IACUC). The approval number for the animal study was GZHU20210301. For hydrodynamic injection, plasmids (Met: PT3EF1aH‐hMet; with Ctnnb1/b‐catenin: PT3EF1aH‐b‐catenin; or PIK3CA: PT3EF1aH‐p110*α* SB transposase: pCMV/SB) were kindly provided by Professor Lijian Hui (Shanghai Institute of Biochemistry and Cell Biology, Chinese Academy of Sciences). Oncogene‐expressing constructs were delivered by hydrodynamic tail vein injection into male mice at 6 weeks of age, as described previously.^[^ ^20^, ^53^, ^54^, ^55^, ^56^ ^]^ Antibodies against Cxcl1 and Il8 were injected into a vein with a concentration of 1 µg/50 µL, once every 2 days for 2 weeks to neutralize the soluble Cxcl1 and Il8 in the liver.

### Human Samples

Human samples were collected at The First Affiliated Hospital, Sun Yat‐sen University. The HCC samples were collected during surgery. Written informed consent was obtained from all patients or their guardians for the use of the biospecimens for research purposes, which were carried out in accordance with the approved guidelines “Use of experimental animals and human subjects.” The approval number of human samples in this study was Approval No. [2022]438. All procedures were approved by the institutional review board of The First Affiliated Hospital, Sun Yat‐sen University.

### Cell Lines

PLC/PRF/5, Hep3B, and Hepa1‐6 cells were obtained from the American Type Culture Collection (ATCC, USA). All cells were authenticated using the short tandem repeat (STR) method and tested negative for mycoplasma.

### Total RNA Isolation, RNA‐seq, and Data Analysis

Total RNA was extracted from the samples using Trizol Reagent (Invitrogen). Purified RNAs were treated with RNase R (Epicenter, 40 U, 37 °C, 3 h), followed by purification with Trizol. Subsequently, using the NEBNext UltraTM RNA Library Prep Kit, RNA‐seq libraries were prepared and subjected to deep sequencing with an Illumina HiSeq 3000 at Seqhealth Technology Co., Ltd., Wuhan, China. Paired‐end 150 bp read length sequencing was performed. Trimmed reads were aligned to the mouse genome (Mm10, Genome Reference Consortium GRCm38) using TopHat v2.0.6.^[^ ^57^ ^]^ FPKM (fragments per kilobase of exon per million fragments mapped) values were calculated by Cufflinks using default parameters for gene expression levels. Differential expression was defined using the indicated fold‐changes and false discovery rate (FDR) 0.05. The Gene Expression Omnibus (GEO) number for the raw data is pending.

### Protein Extraction and Peptide Digestion

The animal studies were performed in compliance with the regulations and guidelines of Guangzhou University. Liver tissues were quickly collected by multispot sampling and pooled after MET/CAT model mice were sacrificed at W0, W2, and W7. The proteins were extracted with SDS lysis buffer (100 mm dithiothreitol, 4% sodium SDS, 100 mm Tris‐HCl, pH 7.6) using mechanical homogenization followed by ultrasonication. The proteins were then denatured and reduced at 95 °C for 5 min. The insoluble debris was removed by centrifugation at 12 000 × *g* for 10 min, and the supernatant was used for proteomic experiments. The protein concentration was determined by a tryptophan‐based fluorescence quantification method. The filter‐aided sample preparation (FASP) method was used for protein digestion as previously described.^[^ ^58^ ^]^ Briefly, 100 µg of protein was loaded into a 10 kDa centrifugal filter tube (Millipore), washed twice with 200 µL of UA buffer (8 m urea in 0.1 m Tris‐HCl, pH 8.5), alkylated with 50 mm iodoacetamide in 200 µL of UA buffer for 30 min in the dark, washed thrice with 100 µL of UA buffer again and finally washed thrice with 100 µL of 50 mm NH_4_HCO_3_. After each of the above steps, the samples were centrifuged at 12 000 × *g* at 25 °C. The proteins were digested with trypsin (Promega Corporation, USA) at an enzyme‐to‐substrate ratio of 1:50 (w/w) in 200 µL of 50 mm NH_4_HCO_3_ at 37 °C for 16 h, and the peptides were eluted by centrifugation. The digested peptides were desalted using C18 Stage Tips and evaporated to dryness in a Speed‐Vac sample concentrator. Finally, the amounts of purified peptides were determined with a NanoDrop (Thermofisher, USA).

### TMT Labeling and High‐pH RP Fractionation

An isobaric labeling experiment was conducted according to the TMT kit instructions. Each channel was labeled with 20 µg peptides. The three samples for W0 were labeled with channels 126, 128N, and 129C; those for W2 were labeled with 127N, 128C, and 130N; and those for W7 were labeled with 127C, 129N, and 130C. Channel 131 was used to label a sample of mixed peptides from W0, W2, and W7. The TMT reagents (0.8 mg) were dissolved in anhydrous acetonitrile (41 µL) and added to the peptides (dissolved in 100 µL of 100 mm TEAB). The labeling reactions were incubated for 1 h at room temperature, and then 8 µL of 5% hydroxylamine was added to the samples for 15 min to quench the reaction. The labeled peptides were pooled, vacuum‐centrifuged to dryness, and subjected to C18 solid‐phase extraction desalting (3 m Empore). High‐pH reversed‐phase (RP) LC was used for peptide fractionation. The labeled peptides were fractionated using a Waters XBridge BEH300 C18 column (150 × 1 mm, OD 5 µm) at a flow rate of 0.2 mL min^−1^ on an Agilent 1200 LC instrument. The mobile phase contained solvent A (10 mm NH_4_COOH, adjusted to pH 10 with NH_3_·H_2_O) and solvent B (90% acetonitrile [ACN], 10 mm NH_4_COOH, adjusted to pH 10 with NH_3_·H_2_O). A 110‐min gradient was set as follows: 1–5% B in 2 min, 5–25% B in 35 min, 25–40% B in 43 min, 40–55% B in 6 min, 55–95% B in 3 min, 95% B for 4 min, 95–1% B in 1 min, and 1% B for 16 min. The eluate was collected every 2 min, and the eluate samples were combined by a concatenation strategy into 25 fractions. The fractions were vacuum‐centrifuged to dryness and then subjected to MS analysis.

### LC‐MS/MS Data Acquisition

LC‐MS/MS analysis was carried out with an EASY‐nLC 1000 liquid chromatograph (Thermo Fisher Scientific) coupled to an Orbitrap Fusion mass spectrometer (Thermo Fisher Scientific). For each fraction, peptides (≈1 µg) were loaded onto an in‐house packed analytical column (75 µm ID × 20 cm, ReproSil‐Pur C18‐Pur, 3 µm, Dr. Maisch GmbH, Ammerbuch, Germany) and subjected to a 120‐min gradient at a flow rate of 300 mL min^−1^. The column was heated to 50 °C using a column compartment to prevent overpressure during LC separation. Mobile phase A consisted of 0.1% formic acid, and mobile phase B consisted of 0.1% formic acid in 100% ACN. The LC gradient was set as follows: 4–28% B in 95 min, 28–40% B in 15 min, 40–100% B in 2 min, and 100% B for 8 min. The spray voltage was set at 2500 V in positive ion mode, and the ion transfer tube temperature was set at 275 °C. The data‐dependent acquisition was performed using Xcalibur software with the profile spectrum data type. The MS1 full‐scan parameters were set to a resolution of 120 000 at *m/z* 200, a lens radio frequency (RF) of 60%, an automatic gain control (AGC) target of 4e5, and a maximum injection time (IT) of 50 ms by Orbitrap mass analyzer (350–1700 *m/z*). “Top‐speed” MS2 scans were generated by higher‐energy C‐trap dissociation (HCD) fragmentation at a resolution of 50 000 at *m/z* 200 with an AGC target of 1e5 and a maximum IT 100 ms. The function “inject ions for all available parallelizable time” was enabled. The isolation window was set at 1.2 *m/z*. The normalized collision energy (NCE) was set at 38%, and the dynamic exclusion time was 40 s. Precursors with charges of 2–6 were included for MS2 analysis.

### Proteomic Database Searching

All mass spectrometric data were analyzed using MaxQuant 1.6.1.0 against the mouse Swiss‐Prot database containing 22 259 sequences (downloaded in August 2019). TMT‐MS2 was chosen for proteomic quantification with a reporter ion mass tolerance of 0.003 Da. Carbamidomethyl cysteine was searched as a fixed modification. Oxidized methionine and protein *N*‐term acetylation were set as the variable modifications. The enzyme specificity was set to trypsin/P. The maximum missed cleavage sites were set to 2. The tolerances of the first search and the main search for peptides were set to 20 and 4.5 ppm, respectively. The minimal peptide length was set to 7. The FDR cutoff for peptides and proteins was set to 0.01. The mass spectrometry proteomics data were deposited to the ProteomeXchange Consortium via the PRIDE partner repository with the dataset identifier PXD017809.

### Proteomic Data Analysis

The Persus, Excel, and R software programs were used for MS data analysis. The correlation matrix, PCA plot, HCA plot, and cluster profile were created in Persus. The significance of the biological process enrichment analysis results for the clusters in Figure 1D was calculated by Fisher's exact test in Persus. The KEGG pathway and GO biological process enrichment analyses were performed on the Database for Annotation, Visualization and Integrated Discovery (DAVID) website, and the adjusted *p*‐values from the Benjamini–Hochberg correction were reported. The protein–protein interaction maps were drawn from the STRING database. The volcano plot was generated in Excel.

### Histology and Immunohistochemistry

Mouse liver samples and human liver cancer samples were fixed overnight in 4% PFA (4 °C) and embedded in paraffin blocks. Immunohistochemistry staining and hematoxylin and eosin staining were performed as previously described.^[^ ^59^, ^60^ ^]^ Tissue sections were stained with the indicated antibodies. The tissue sections were quantitatively scored according to the percentages of positive cells and the staining intensity and determined the localization of the signals as described previously.^[^ ^59^, ^60^ ^]^

### Measuring Reactive Oxygen Species in Liver Tissue

The ROS level of liver tissue was measured using 2,7‐dichlrofluorescein diacetate (DCFDA), which was converted to highly fluorescent DCF by cellular peroxides (including hydrogen peroxide). The assay was performed as described before.^[^ ^61^, ^62^ ^]^ In brief, 1% tissue homogenates were prepared in ice‐cold 40 mm Tris–HCl buffer (pH 7.4). Samples were further diluted to 0.25% with the same buffer and placed on ice. Each sample was divided into two equal fractions (2 mL each). To one fraction, 40 µL of 1.25 mm DCFDA in methanol was added for ROS measurement. The same volume of methanol was added to the other fraction as a control for sample autofluorescence. All samples were incubated for 15 min in a 37 °C water bath. The fluorescence was determined at 488 nm excitation and 525 nm emission using a Bio‐Rad fluorometer.

### Quantification of 3PG, 3PHP, and Ser by LC‐MS

The metabolites were extracted from mouse liver tissues (≈20 mg) by homogenization in 500 µL 80% methanol followed by centrifugation in 12 000 g for 15 min at 4 °C. The supernatant (≈450 µL) was transferred into new tubes for vacuum freeze drying. The samples were resolved in 100 µL 60% ACN and 1 µL metabolites were used for quantification of 3PG, 3PHP, and Ser by a targeted multiple reaction monitoring (MRM) mass spectrometry method. For metabolites extraction from mouse serum, 80 µL of serum was mixed with 320 µL of acetonitrile by vortexing for 1 min, and then the sample was centrifuged at 20 000 g for 10 min at 4 °C following which 300 µL of the supernatant was transferred to a new tube for lyophilization and the next MS analysis. An UltiMate 3000 UHPLC system equipped with a TSQ Quantiva mass spectrometer (Thermo Scientific) was used for this analysis. The LC gradient used an ACQUITY UPLC BEH Amide column (2.1 × 100 mm, 1.7 µm) at a flow rate of 0.3 mL min^−1^. Solvent A was 15 mm NH4HCO3 in 100% water (pH 9, adjusted by NH3·H2O) and solvent B was 15 mM NH4HCO3 in 90% ACN. A gradient of 8.7 min was set as follows: 90% B to 40% B for 1.5 min; 40% B for 3.5 min; 40% B to 90% B for 0.2 min; 90% B for 3.5 min. The column compartment was set at 50 °C and the autosampler was set at 4 °C. The MS analysis was conducted with an H‐ESI source operating in negative mode. The MS parameters were set as follows: Spray voltage, 2500 V; Sheath gas (Arb), 40; Aux gas, 8; Sweep gas, 1; Ion transfer tube temperature, 350 °C; Vaporizer temperature, 350 °C. Both Q1 and Q3 resolutions were set at 0.7 amu. Collision‐induced dissociation used argon as the CID gas at 1.5 mTorr. Three standards (3‐phosphoglyceric acid disodium salt, P8877, Sigma‐Aldrich; Hydroxypyruvic acid phosphate lithium salt, 0 2711, Sigma‐Aldrich; l‐Serine, S4500, Sigma‐Aldrich) were used for SRM parameter optimization and target peak determination. The SRM parameters for the three metabolites were set as follows: Name‐Precursor (*m/z*)‐Product (*m/z*)‐CE (V) ‐RF lens (V); 3PG‐185‐79‐31‐52; 3PHP‐183‐79‐24‐61; Ser‐104‐74‐10‐50. Data analysis was performed with Xcalibur software.

### Immunoprecipitation, Immunoblotting Analysis, and LC‐MS

Immunoprecipitation was performed with lysates from the indicated cultured cells and was followed by IB with corresponding antibodies. Briefly, after trypsinization, cells were harvested and washed twice with cold PBS. The cell pellets were resuspended and lysed in a lysis buffer (Millipore). Then, the lysates were sonicated with a Covaris S220 to destroy chromatin and centrifuged at 15 000 × *g* to remove the cell debris. The protein concentration was determined using a BCA Protein Assay Kit (Pierce) according to the manufacturer's instructions. A total of 100 mg of protein was incubated with the indicated antibodies overnight and then mixed with protein A or protein G‐agarose beads (Millipore), respectively. The immunocomplexes were collected by centrifugation at 1000 × *g*, resolved by SDS–PAGE, and subsequently transferred to PVDF membranes (Millipore). The blots were blocked with 5% nonfat milk and then incubated with primary antibodies and HRP‐conjugated secondary antibodies. The blots were developed using SuperSignal West Pico (Thermo Scientific) and detected with a Tanon 6200 Luminescent Imaging Workstation. For LC‐MS of the immunocomplexes, the proteins were resolved by SDS‐PAGE and subjected to in‐gel digestion by trypsin as the widely used protocol.^[^ ^63^ ^]^ The resulting peptides were separated by an LC gradient for 2 h and analyzed on a Q Exactive HF mass spectrometer (Thermo Fisher Scientific). The MS data were searched against the mouse database using the MaxQuant software. The iBAQ (intensity‐based absolute‐protein‐quantification) intensity was used for comparison between samples.

### DNA Constructs and Stable Cell Lines

PCR‐amplified PHGDH and cMyc were cloned into pCDH, pCDH‐3′Flag, or 3′HA. The target sequence of PHGDH is listed in Table S1, Supporting Information, as a nontargeting sequence was used as a negative control. The cMyc reporter plasmid was the same as those used in a previous study.^[^ ^64^ ^]^ Cells cultured in Dulbecco's modified Eagle's medium (DMEM) or Roswell Park Memorial Institute (RPMI) 1640 medium were incubated with a retrovirus encoding shRNA or pCDH for 8 h. 48 h after infection, the cells were selected by culture with 2 mg mL^−1^ puromycin.

### Cell Culture and Transfection

Cells were cultured as described previously.^[^ ^59^ ^]^ The above cells were maintained in DMEM or RPMI medium supplemented with 10% fetal bovine serum (FBS). Cell transfection was performed with Lipofectamine 3000 (Invitrogen, USA) according to the manufacturer's instructions.

### Subcellular Fractionation Analyses

PLC/PRF/5 and Hep3B cells were collected and washed three times with cold PBS. Nuclear or cytosolic fractions were prepared using a Nuclear Extract Kit (Active Motif, USA).

### Immunofluorescence Staining

Cells were fixed and incubated with anti‐PHGDH and anti‐cMyc antibodies, Alexa Fluor dye‐conjugated secondary antibodies, and DAPI according to standard protocols. Liver tissues were fixed in 4% formaldehyde and embedded in optimum cutting temperature (OCT) compound (Thermo) after dehydration by 30% sucrose solution. The tissue sections were washed in PBS, 0.3% Triton X‐100, and blocked in PBS with 10% normal goat serum followed by incubation with anti‐Ly6G, anti‐CD11b, or anti‐CD68 antibodies. After washing, samples were incubated with Alexa Fluor dye‐conjugated secondary antibodies and DAPI. The imaging was performed on an Olympus FV3000 confocal laser scanning microscope (Olympus).

### Orthotopic Model of Liver Cancer

*Phgdh*‐depleted Hepa 1–6 cells rescued with rPhgdh‐WT, rPhgdh‐dACT, or rPhgdh‐dACT & Cxcl1/Il18 were resuspended in PBS and mixed with an equal volume of growth factor‐reduced Matrigel (BD Biosciences). Randomized 6‐week‐old male athymic nude mice were anesthetized, and 2 × 10^[^ ^5^ ^]^ cells in a volume of 50 µL were injected into the medial lobe of the livers. After inoculation for 10 days, animals were euthanized, and the livers were dissected and fixed in 4% paraformaldehyde.

### Tumor‐Infiltrating Immune Cell Isolation and Flow Cytometry

Mice were killed by cervical dislocation and sterilized in 75% alcohol for 3 min. The mouse limbs were pinned to the foam board, the mouse skin was dissected with scissors and forceps, and the intact tumor was carefully harvested. The mouse tumors were soaked in PBS to remove superficial blood. Then the tumor tissue was minced, placed in a 15 mL centrifuge tube, 8 mL of digestion solution was added, and shacked at 37 °C for 1 h. Large pieces of tissue were removed by centrifugation at 50 g for 1 min and the supernatant was collected. Centrifuged at 600 g for 8 min, removed the supernatant, and collected the cell pellet. Prepared 37.5% percoll cell separation medium. Resuspend the cell pellet in 10 mL percoll per tube. Centrifuged at 500 g for 30 min at 22 °C, removed the supernatant, and collected the cell pellet. Added 1 mL of erythrocyte lysing solution to the cell pellet, pipetting evenly, and placed on ice for 1–3 min to lyse the erythrocytes. The lysis was terminated by adding 9 mL of PBS, centrifuged at 600 g for 8 min, and the cell pellet was collected. The cell pellet was resuspended in 250 µL PBS+2% FBS solution, the antibody was added, and stained on ice for 30 min in the dark. Flow cytometry analysis was then performed.

### Neutrophil Transwell Assays

Neutrophils were isolated according to the methods described previously^[^ ^65^ ^]^ and cultured with or without CXCL1/IL8 neutralizing antibodies (1:50 by volume). Transwell assays were performed on a 24‐well plate with inserts (BD Biosciences) according to the manufacturer's instructions. Briefly, 5 × 10^[^ ^4^ ^]^ of neutrophils were cultured in the upper chamber and allowed to migrate for 36 h before fixation for crystal purple staining. The lower culture medium was obtained by culturing PLC/RF/5 and Hep3B in DMEM medium containing 10% FBS for 36 h and then used for the cell migration transwell assay.

### PHGDH Expression Analysis of the Previous Published Clinical Datasets

The TCGA data (Figure 7A) was downloaded from https://gdac.broadinstitute.org/. The protein data (Figure 7B) were from two proteomic datasets of HCC, Gao et al. 2019, *Cell*,^[^ ^66^ ^]^ and Jiang et al. 2019, *Nature*.^[^ ^67^ ^]^

### Survival Analysis and Gene Expression Correlation Analysis

Survival analysis was performed in GraphPad Prism software, and the log‐rank test *p*‐values were reported. TCGA‐LIHC gene expression and survival data for PHGDH and cMyc were downloaded from www.tcgaportal.org. Survival plots for PHGDH and cMyc were reconstructed in GraphPad with median cutoffs. To create Figure 7J, a combined survival analysis of PHGDH and cMyc was performed. Given the different expression levels of the two proteins, the abundances of PHGDH and cMyc were ranked and then summed the ranks as the combined abundance (PHGDH+cMyc) levels to enable comparison.

### AAV Virus In Vivo Delivery

For AAV virus infection, 2 × 10^[^ ^11^ ^]^ genomic particles of AAV‐Vec, AAV‐Phgdh‐WT, AAV‐Phgdh‐ED, and AAV‐Phgdh‐dACT were reconstituted in 200 µL PBS and injected intravenously through tail veil injection with BD Ultra‐Fine Insulin Syringes.

### Statistical Analysis

All data are presented as the mean ± SD unless stated otherwise. Statistical calculations were performed using GraphPad Prism software (version 8.0.2). Unpaired Student's *t*‐test and one‐way ANOVA followed by multiple comparison tests (Dunnett's or Tukey's method) were used to calculate statistical probability in this study. Two‐tailed log‐rank test was used to analyze survival data. The number of animals used for each experiment is indicated in the figure legends.

## Conflict of Interest

The authors declare no conflict of interest.

## Supporting information

Supporting Information

Supporting Table 1

Supporting Table 2

## Data Availability

The data that support the findings of this study are available from the corresponding author upon reasonable request.
