# Supplementary material for: Elevated Nuclear PHGDH Synergistically Functions with cMyc to Reshape the Immune Microenvironment of Liver Cancer
Source: Adv Sci (Weinh). 2023 Apr 20;10(17):2205818. doi: 10.1002/advs.202205818 (PMC10265107; doi:10.1002/advs.202205818)
Supplement: Supplementary file 1 — Supporting Information [file ADVS-10-2205818-s002.pdf]

ADVANCED SCIENCE
Open Access

Supporting Information

for *Adv. Sci.*, DOI 10.1002/advs.202205818

Elevated Nuclear PHGDH Synergistically Functions with cMyc to Reshape the Immune Microenvironment of Liver Cancer

Hongwen Zhu, Hua Yu, Hu Zhou, Wencheng Zhu* and Xiongjun Wang**

Supplementary figures legends

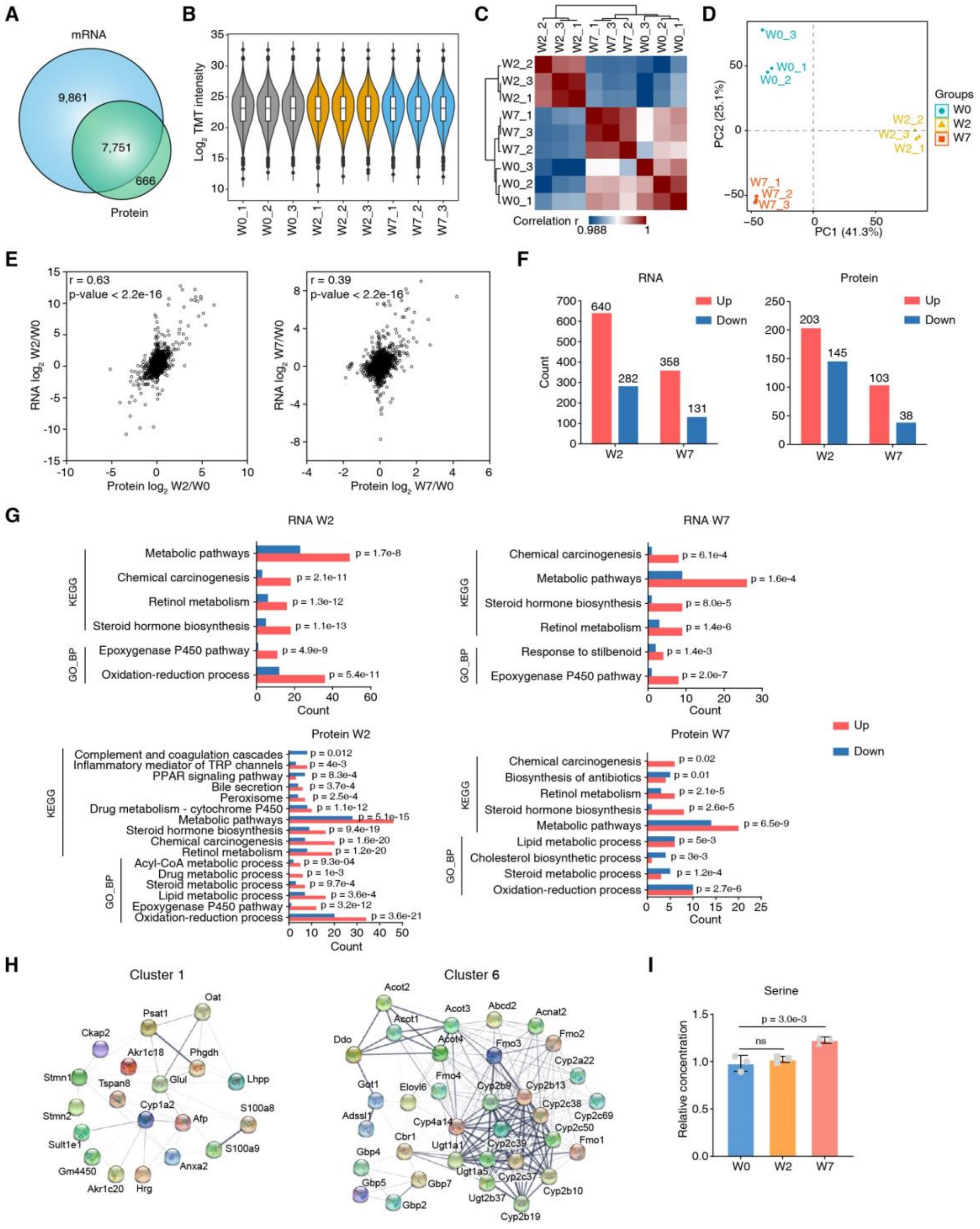

Fig. S1. Related to Fig. 1. Overall quality and uniformity of the proteomic and RNA profile data.

(**A**) Overlap of the identified mRNA and proteins at the gene level. A total of 17,612 RNAs and 8,417 proteins were quantified at all three time points.

(**B**) Boxplot of the log2 transformed TMT intensity of the proteomic data.

(**C**) Correlation matrix of the proteomic data. The three repeats for the same time point were clustered into a subgroup.

(**D**) Principal component analysis of the proteomic data. The three repeats for the same time point

were clustered together.

(**E**) Correlations between RNA and protein levels at W2 (C) and W7 (D) compared to W0. The Pearson correlation coefficient and p-value were calculated.

(**F**) Counts of up- and down-regulated genes and proteins with p-value < 0.05 and FC > 2 or < 0.5. Red indicates upregulation, and blue indicates downregulation.

(**G**) KEGG pathway and GO biological process enrichment analysis results for RNA sequences and proteins regulated in W2 vs. W0 and in W7 vs. W0. The x-axis represents the gene count. The enrichment analysis was performed in the DAVID, and the adjusted p-value of each item is labeled on the right side.

(**H**) Protein-protein interaction maps of the proteins in Cluster 1 and Cluster 6 were drawn using the STRING database. The line thickness between every two proteins indicates the strength of the interaction.

(**I**) The serine levels in serum were measured in mice after MET/CAT induction at different time points. (Mean ± SD, two-tailed Student's t-test, n = 3).

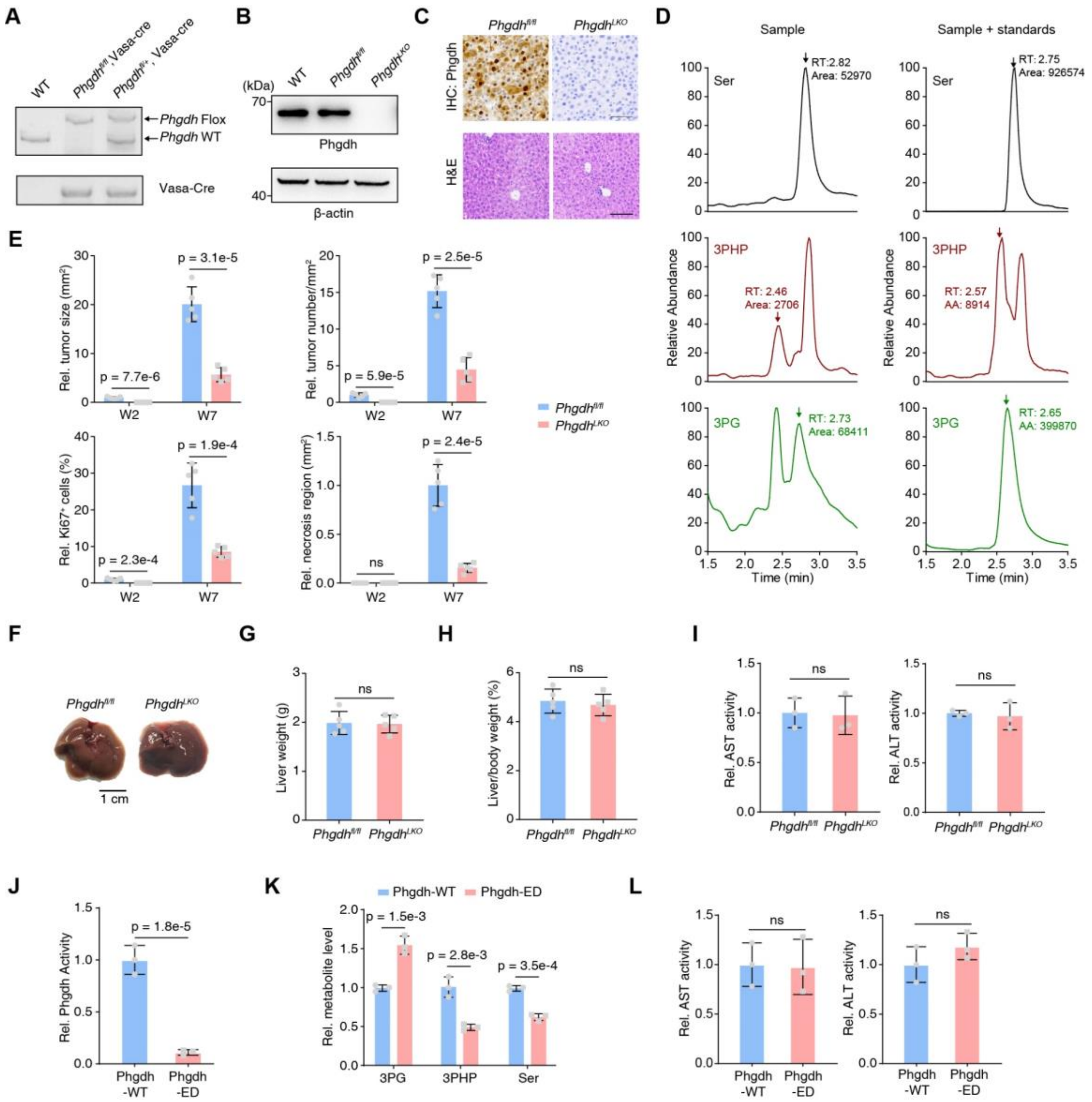

Fig. S2. Related to Fig. 2. Hepatic loss of *Phgdh* enhances mouse survival after MET/CAT-driven hepatocarcinogenesis.

(**A**) Genotypes were determined by PCR using liver genomic DNA.

(**B**) Phgdh protein in liver tissues of *Phgdh*$^{fl/fl}$ and *Phgdh*^{LKO} mice was detected by immunoblotting. Loading control, β–actin.

(**C**) IHC staining of Phgdh and H&E staining using liver sections from the indicated mice at 6-week-old.

(**D**) The targeted MRM quantification method was used to measure the relative changes of three metabolites (3PG, 3PHP and Ser). The sample was separated by an ACQUITY UPLC® BEH Amide column and analyzed on a TSQ Quantiva MS (left panel). To further ascertain which is the target peak, the three standard substances was added to the above sample and analyzed again using the same LC-MS method to see which peak was increased markedly in area (right panel).

(**E**) The tumor size, number of tumor nodule, percentage of Ki67-positive cell and necrosis region were measured in *Phgdh*$^{fl/fl}$ and *Phgdh*^{LKO} livers at W9 after MET/CAT injection. (Mean ± SD, two-tailed Student's t-test, n = 5 per group).

(**F**) Macroscopic images mouse (*Phgdh*$^{fl/fl}$ and *Phgdh*^{LKO}) livers at 6-week-old.

(**G**) The liver weight of the indicated mice at 6-week-old were measured. (Mean ± SD, two-tailed Student's t-test, n = 3; ns, not significant.)

(**H**) The liver/body weight ratios of the indicated mice at 6-week-old were measured. (Mean ± SD, two-tailed Student's t-test, n = 3; ns, not significant.)

(**I**) The serum ALT and AST levels were measured in the indicated mice at 6-week-old without MET/CAT induction. (Mean ± SD, two-tailed Student's t-test, n = 3)

(**J**) Phgdh was immuno-precipitated from the indicated livers and incubated with its substrate 3PG and NAD^+ for testing the enzymatic activity. The protein levels were adjusted to the same extent. (Mean ± SD, two-tailed Student's t-test, n = 3)

(**K**) The relative levels of three metabolites (3PG, 3PHP and Ser) were measured in liver samples of *Phgdh*^{LKO} mice receiving rPhgdh-WT or rPhgdh-ED AAV. (Mean ± SD, two-tailed Student's t-test, n = 6.)

(**L**) The serum ALT and AST levels were measured in the indicated mice at 6-week-old. (Mean ± SD, two-tailed Student's t-test, n = 3).

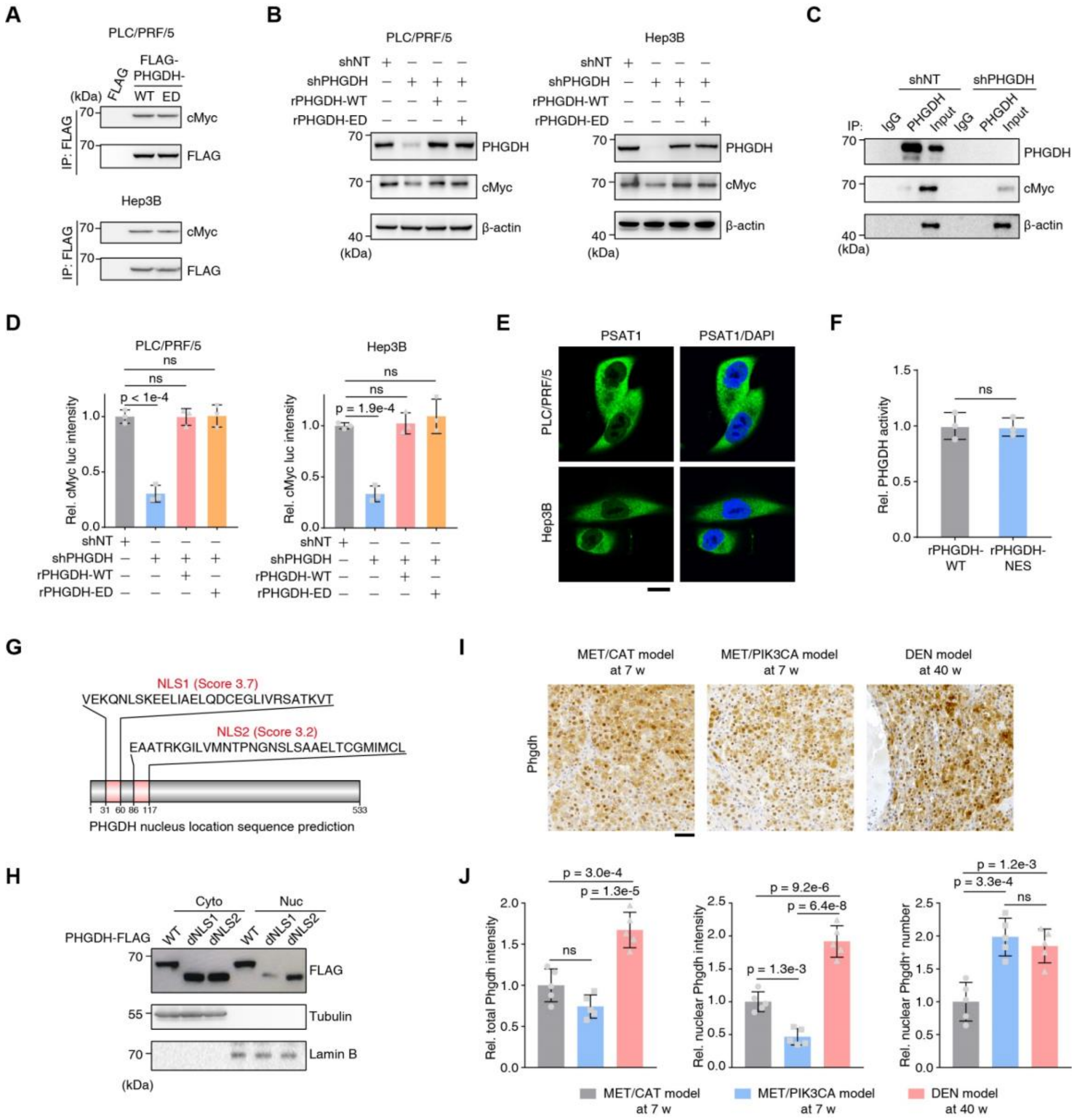

Fig. S3. Related to Fig. 3.

(**A**) Co-IP was performed with an antibody against FLAG in PLC/PRF/5 and Hep3B (FLAG tagged

PHGDH-WT and -ED) cells. An antibody against cMyc was used to detect the association between FLAG-PHGDH and cMyc.

(**B**) PLC/PRF/5 or Hep3B cells stably expressing shNT or shPHGDH were rescued with rPHGDH-WT or rPHGDH-ED. The abundance changes of PHGDH and cMyc were analyzed by immunoblot.

(**C**) Co-IP assay was conducted to detect the changes of interaction between PHGDH and cMyc in PHGDH KD PLC/PRF/5 cells compared to control cells.

(**D**) cMyc transactivation in PHGDH-depleted PLC/PRF/5 or Hep3B cells rescued with rPHGDH-WT or rPHGDH-ED was measured with a Dual-Luciferase® Reporter Assay System according to the manual.

(**E**) Immunofluorescence imaging of PSAT1 in two cell lines.

(**F**) Phgdh was immuno-precipitated from the indicated cells and incubated with its substrate 3PG

and NAD+ for testing the enzymatic activity. The protein levels were adjusted to the same extent. (Mean ± SD, two-tailed Student's t-test, n = 3).

(**G**) Prediction of NLS in PHGDH by cNLS Mapper. Two sequences and their corresponding NLS score were shown.

(**H**) Evaluation of two predicted NLS in nuclear import of PHGDH by deletion of each NLS.

(**I**) IHC staining of Phgdh in three models of hepatocarcinogens in mouse. Scale bar, 50 µm.

(**J**) IHC intensity of total Phgdh and nuclear Phgdh, and nuclear Phgdh-positive cell number were measured in different models. (Mean ± SD, one-way ANOVA followed by Tukey's multiple comparisons test, n = 5 per group)

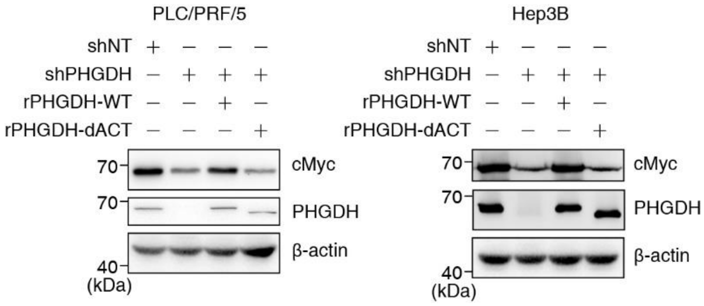

Fig. S4. Related to Fig. 4. PLC/PRF/5 or Hep3B cells stably expressing shNT or shPHGDH were rescued with rPHGDH-WT or rPHGDH-dACT.

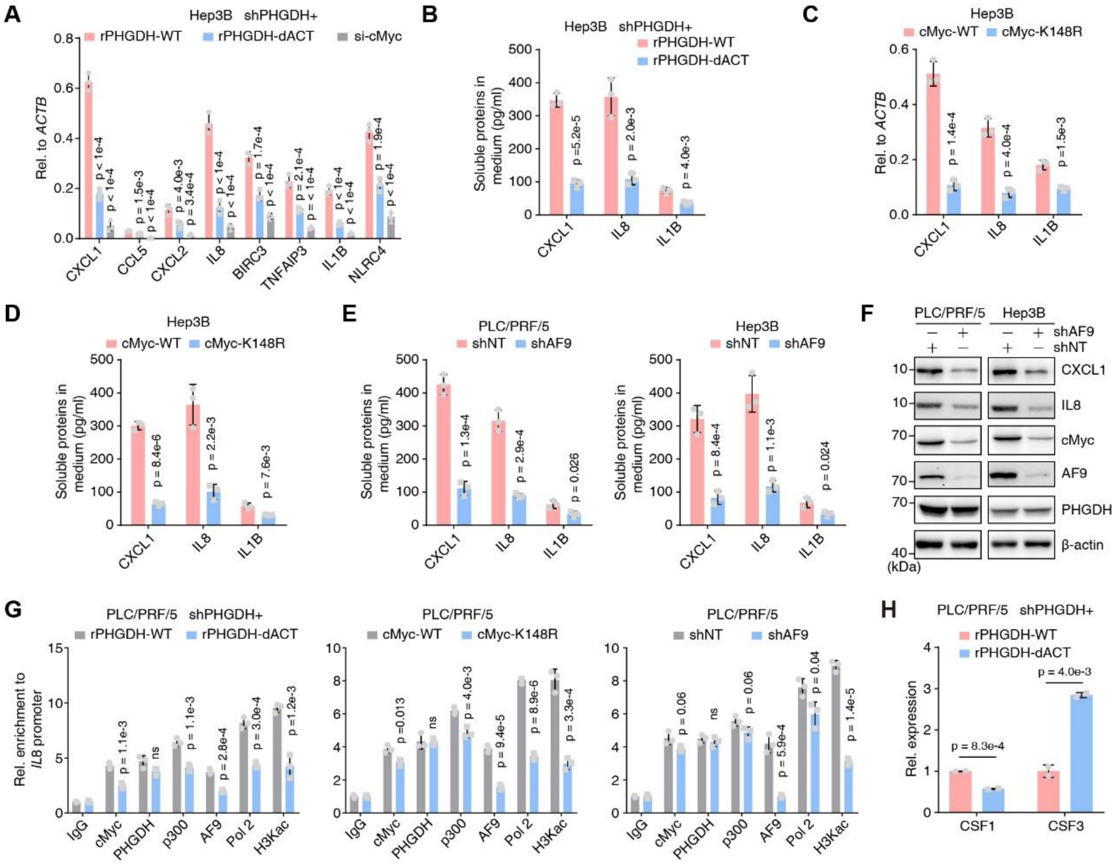

Fig. S5. Related to Fig. 5.

(**A**) qRT-PCR validated the top up-regulated genes in the indicated cells from Figure 5C. (Mean ± SD, one-way ANOVA followed by Dunnett's multiple comparisons test, n = 3).

(**B**) ELISA examined the concentration of CXCL1/IL8 and IL1B in the medium culturing PHGDH-depleted Hep3B cells, which were rescued with rPHGDH-WT or rPHGDH-dACT.

(**C**) qRT-PCR validated CXCL1/IL8 and IL1B genes using Hep3B cells expressing WT or K148R mutant Myc. (Mean ± SD, two-tailed Student's t-test, n = 3).

(**D**) ELISA examined the concentration of CXCL1/IL8 and IL1B in the medium culturing Hep3B cells expressing WT or K148R mutant Myc. (Mean ± SD, two-tailed Student's t-test, n = 3).

(**E**) ELISA examined the concentration of CXCL1/IL8 and IL1B in the medium culturing PLC/PRF/5 or Hep3B cells expressing shNT or shAF9. (Mean ± SD, two-tailed Student's t-test, n = 3).

(**F**) Immunoblotting analysis of CXCL1/IL8, PHGDH, AF9 and cMyc was performed using the indicated cells and antibodies.

(**G**) ChIP analysis of PHGDH, cMyc, p300, RNA Pol II, AF9 and H3Kac on CXCL1 gene promoter was performed using indicated cells. IgG was used as a blank control. (Mean ± SD, two-tailed Student's t-test, n = 3).

(**H**) qRT-PCR validated CSF1/3 genes using PHGDH-depleted PRF/PLC/5 cells expressing rPHGDH-WT or rPHGDH-dACT. (Mean ± SD, two-tailed Student's t-test)

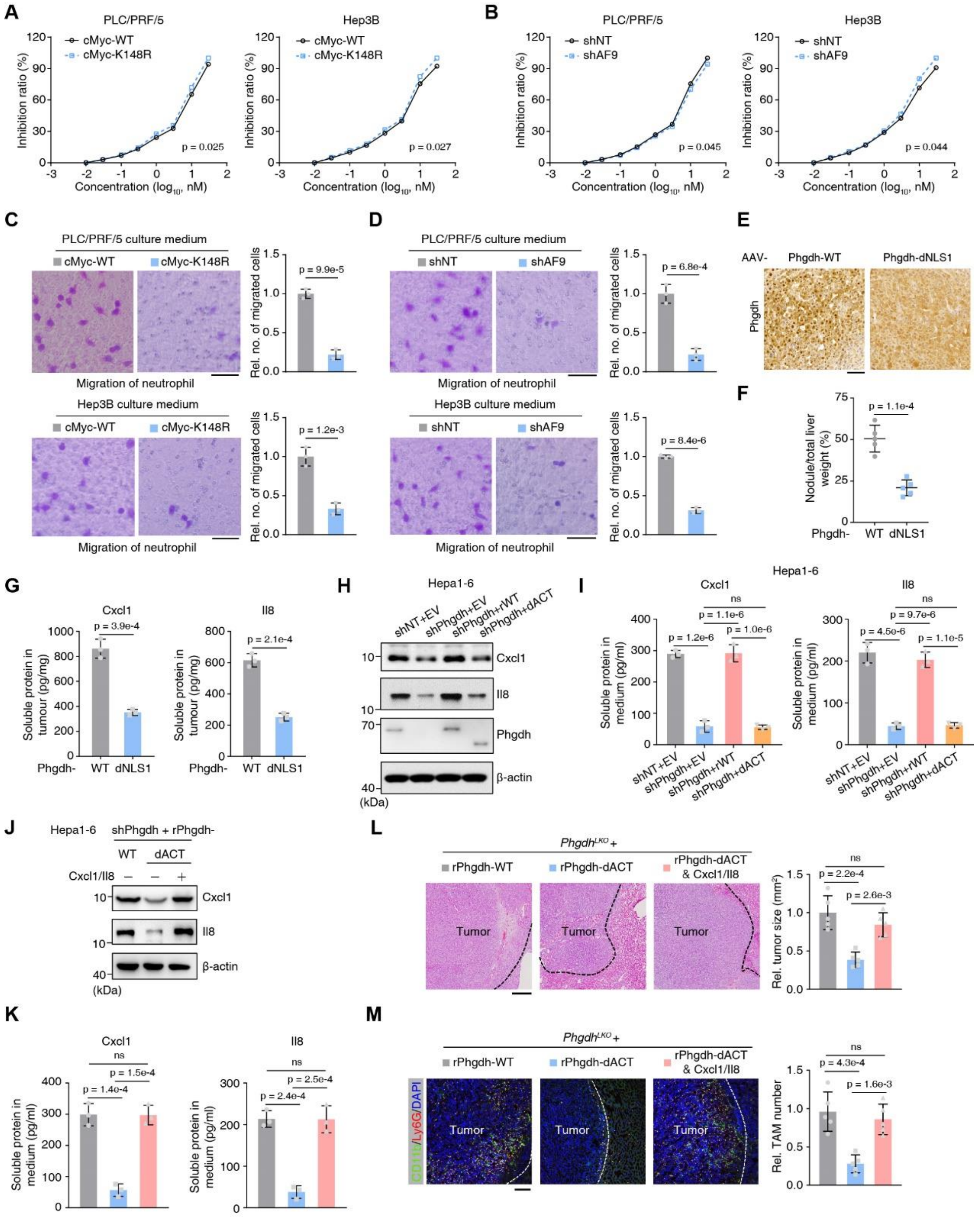

Fig. S6. Related to Fig.6.

(**A**) Sorafenib inhibition was evaluated by trypan blue staining in PLC/PRF/5 or Hep3B cells expressing WT or K148R mutant cMyc. (two-way ANOVA).

(**B**) Sorafenib inhibition was evaluated by trypan blue staining in PLC/PRF/5 or Hep3B cells expressing shNT or shAF9. (two-way ANOVA).

(**C**) Neutrophil recruitment was evaluated by cell migration, which was performed by placing the medium from culturing PLC/PRF/5 or Hep3B cells expressing WT or K148R mutant cMyc in the lower well and neutrophil cells in the upper transwell chamber. Scale bars: 20 µm. (Mean ± SD, two-tailed Student's t-test, n = 3).

(**D**) Neutrophil recruitment was evaluated by cell migration, which was performed by placing the

medium from culturing PLC/PRF/5 or Hep3B cells expressing shNT or shAF9 in the lower well and neutrophil cells in the upper transwell chamber. Scale bars: 20 μm. (Mean ± SD, two-tailed Student's t-test, n = 3).

(**E**) IHC staining of Phgdh in mouse liver sections at W9 after *Phgdh*^{LKO} mice receiving AAV treatments (Vec, Phgdh-WT or Phgdh-dNLS1). Scale bar, 100 μm.

(**F**) Tumor nodule/total liver weight ratios of *Phgdh*^{LKO} mice after indicated treatments was measured at W9 post MET/CAT injection. (Mean ± SD, two-tailed Student's t-test, n = 5).

(**G**) ELISA examined the concentration of Cxcl1 and Il8 in the tumor lysates from the indicated livers. (Mean ± SD, two-tailed Student's t-test, n = 3).

(**H**) Expression of Cxcl1 and Il8 was measured by western blotting in the indicated Hepa 1-6 cells.

(**I**) ELISA examined the concentration of Cxcl1 and Il8 in the medium from the indicated Hepa 1-6 cells. (Mean ± SD, one-way ANOVA followed by Tukey's multiple comparisons test, n = 3)

(**J**) Hepa 1-6 cells stably expressing shPhgdh were rescued with rPhgdh-WT, rPhgdh-dACT, or rPhgdh-dACT with Cxcl1/Il8 overexpression.

(**K**) ELISA examined the concentration of Cxcl1 and Il8 in the medium from the indicated Hepa 1-6 cells from (J). (Mean ± SD, one-way ANOVA followed by Tukey's multiple comparisons test, n = 3).

(**L**) The indicated Hepa 1-6 cells from (J) were orthotopically transplanted into livers of *Phgdh*^{LKO} mice. H&E staining of mouse liver sections was performed ten days after transplantation. Tumor region was outlined with dark dash line. The relative tumor size was measured. (Mean ± SD, one-way ANOVA followed by Tukey's multiple comparisons test, n = 5 per group).

(**M**) IF staining of Ly6G^{+} CD11b^{+} TANs in mouse liver sections at day 10 after orthotopically transplantation. The relative number of TAN was calculated. Scale bars: 100 μm. (Mean ± SD, one-way ANOVA followed by Tukey's multiple comparisons test, n = 5 per group).

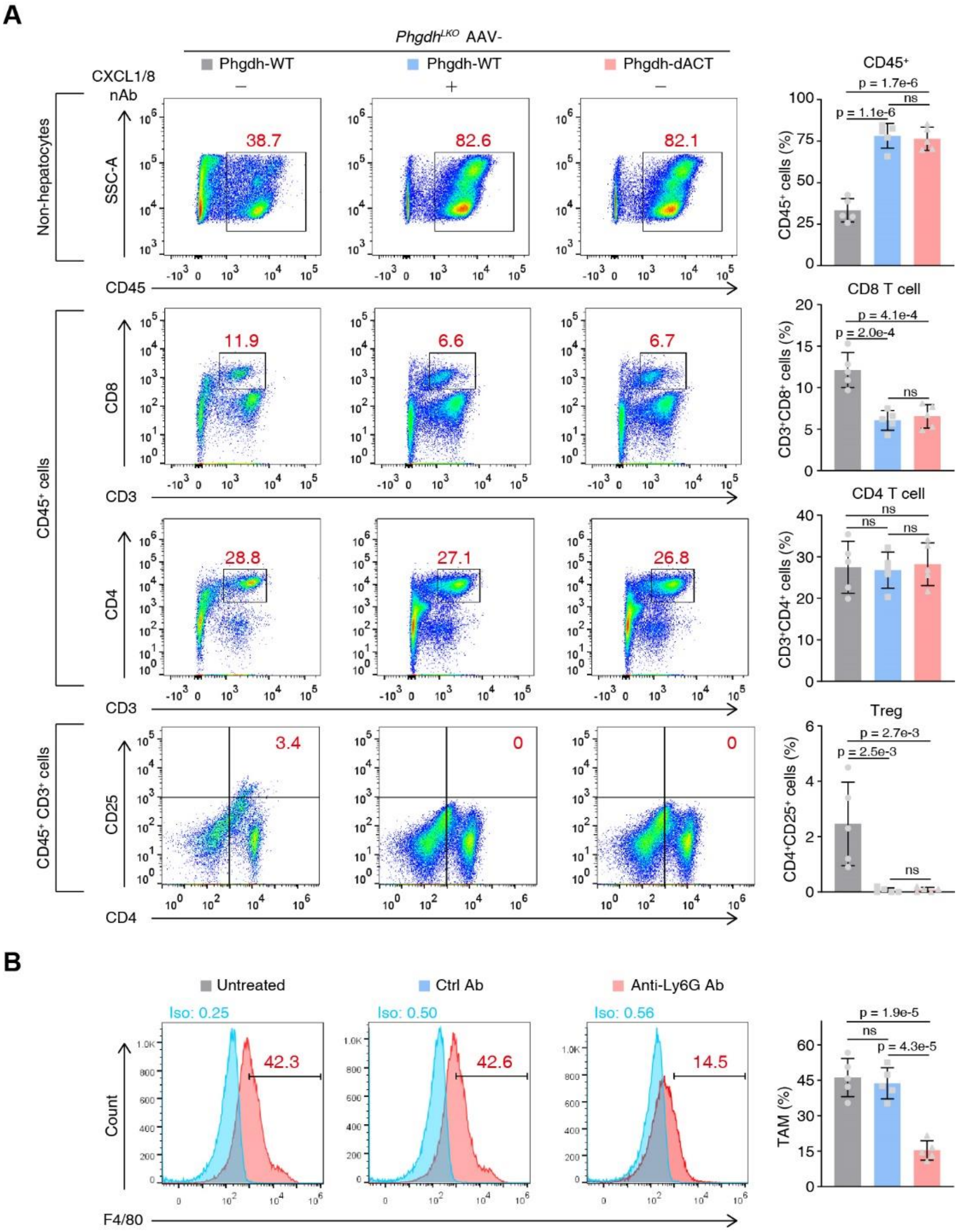

Fig. S7. Related to Fig.6.

(**A**) Representative flow cytometry data of leukocytes (CD45$^+$), CD8T (CD3$^+$ CD8$^+$), CD4T (CD3$^+$ CD4$^+$) and Treg (CD3$^+$ CD4$^+$ CD25$^+$) from the indicated livers. (Mean ± SD, one-way ANOVA followed by Tukey's multiple comparisons test, n=5).

(**B**) Representative flow cytometry data of TAMs (F4/80$^+$) from mouse livers after the indicated treatments. (Mean ± SD, one-way ANOVA followed by Tukey's multiple comparisons test, n=5)

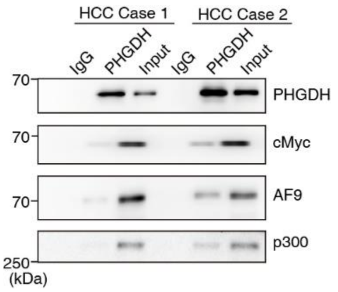

Fig. S8. Related to Fig. 7

Co-IP assay was conducted to detect the interaction between PHGDH and cMyc/AF9/p300 in human HCC samples.

Supplemental Materials and Methods

qRT-PCR. Total RNA was prepared from cell samples using TRIzol (Invitrogen, USA) according to the manufacturer's protocol. Reverse transcriptase PCR was performed using M-MLV reverse transcriptase (Promega, USA). qRT-PCR was performed using SYBR® Premix Ex Taq™ (Takara, Japan) and 300 nmol/L of each primer. Amplification was performed with a 7500 Fast Real-Time PCR Systems (Applied Biosystems, USA) according to the manufacturer's protocol. The data were normalized to the expression of the control gene (β-actin) for each experiment. The data are presented as the mean ± SD from three independent experiments. The sequences of the primer pairs used for qRT-PCR are listed in Supplemental Table 2.

GST pull-down assay. Purified proteins, GST-ACT (1 μg) and His-cMyc (1 μg) were incubated at room temperature for 2 hours, and then GST beads were added to pull down the GST-tagged proteins for an additional 2 hours. The GST beads were washed with lysis buffer (Millipore) three times for 5 min each time. Then, the GST beads bound with proteins were boiled for 8 min after adding SDS loading buffer.

Luciferase reporter gene assay. The transcriptional activation of cMyc in liver cancer cells was measured using a Dual-Luciferase Assay Kit (Promega) on a GloMax 20/20 luminometer (Promega, E1910) following the manufacturer's instructions. In detail, the cells were plated in triplicate at a density of 2×10^4 cells/well in a volume of 500 μl in separate 24-well microtiter plates. After transfection with the indicated plasmids for 48 hours, the cells were washed with cold PBS and lysed with lysis buffer. The relative levels of luciferase activity were normalized to the levels in untreated cells and to the levels of Renilla luciferase activity of the control plasmid in each group.

ChIP assay. For ChIP experiments, cells were fixed in 1% formaldehyde for 10 min for pull-down of PHGDH, RNA Pol II, AF9, cMyc, p300 and H3Kac. The cells were rotated in cold lysis buffer (10 mM Tris-HCl pH 7.4, 10 mM NaCl, 3 mM $MgCl_2$, 0.5% NP40, supplemented with freshly prepared PMSF and protease inhibitor cocktail) for 30 min. Then, the nuclear pellets were resuspended in RIPA buffer (300 mM NaCl, 3 mM EDTA, 1% NP40, 0.5% sodium deoxycholate, 0.1% SDS, 100 μg/ml BSA, 50 mM Tris-HCl pH 7.5) and sonicated with a Covaris S220 to yield DNA fragments of approximately 200–500 bp. A ChIP-grade antibody was

incubated with 30 μl of protein G Dynabeads (Thermo Fisher) for 4 hours. Then, the DNA fragments were coimmunoprecipitated with the specific antibody-conjugated protein G beads at 4°C overnight. The DNA was purified with a MinElute PCR Purification Kit (Qiagen, Germany). The promoter regions of target genes were amplified and quantified by qRT-PCR using SYBR Green (Takara, Japan) on an ABI 7500 Fast system. For PCR, 0.02 ng of the immunoprecipitated DNA and 2 ng of the total DNA were used in a 20 μl reaction. The results from each immunoprecipitation were normalized to the respective inputs. The primers are listed in Supplemental Table 2.

Cell proliferation assay. Cell proliferation was analyzed with CCK-8 assay. Cells were seeded into 96-well plates (500 cells/well), then incubated at 37 °C for the next 6 days. CCK-8 assay was performed conforming to the manufacturer's instructions every two days. The OD value for each well was measured at 450 nm with a reference at 650 nm using a microtiter plate reader (Becton Dickinson).

Sorafenib sensitivity assays. To determine the sensitivity of liver cancer cells to Sorafenib, 2×10^4 of live tumor cells were seeded in 96 well plates and treated with different concentrations of Sorafenib (from 0 to 100 nM). After 72 hours, the cell viability was determined by Trypan blue staining assay and inhibition efficiency was calculated using Graphpad Prism 7.
